# A Nomogram for Preoperatively Predicting the Ki-67 Index of a Pituitary Tumor: A Retrospective Cohort Study

**DOI:** 10.3389/fonc.2021.687333

**Published:** 2021-05-31

**Authors:** Xiangming Cai, Junhao Zhu, Jin Yang, Chao Tang, Feng Yuan, Zixiang Cong, Chiyuan Ma

**Affiliations:** ^1^ School of Medicine, Southeast University, Nanjing, China; ^2^ School of Medicine, Nanjing Medical University, Nanjing, China; ^3^ Department of Neurosurgery, Jinling Hospital, Nanjing, China; ^4^ School of Medicine, Nanjing University, Nanjing, China; ^5^ School of Nanjing Medicine, Southern Medical University, Guangzhou, China

**Keywords:** age, Ki-67, nomogram, pituitary tumors, primary-recurrence subtype, prolactin

## Abstract

**Background:**

The Ki-67 index is an indicator of proliferation and aggressive behavior in pituitary adenomas (PAs). This study aims to develop and validate a predictive nomogram for forecasting Ki-67 index levels preoperatively in PAs.

**Methods:**

A total of 439 patients with PAs underwent PA resection at the Department of Neurosurgery in Jinling Hospital between January 2018 and October 2020; they were enrolled in this retrospective study and were classified randomly into a training cohort (n = 300) and a validation cohort (n = 139). A range of clinical, radiological, and laboratory characteristics were collected. The Ki-67 index was classified into the low Ki-67 index (<3%) and the high Ki-67 index (≥3%). Least absolute shrinkage and selection operator algorithm and uni- and multivariate logistic regression analyses were applied to identify independent risk factors associated with Ki-67. A nomogram was constructed to visualize these risk factors. The receiver operation characteristic curve and calibration curve were computed to evaluate the predictive performance of the nomogram model.

**Results:**

Age, primary-recurrence subtype, maximum dimension, and prolactin were included in the nomogram model. The areas under the curve (AUCs) of the nomogram model were 0.694 in the training cohort and 0.658 in the validation cohort. A well-fitted calibration curve was also generated for the nomogram model. A subgroup analysis revealed stable predictive performance for the nomogram model. A correlation analysis revealed that age (R = −0.23; *p* < 0.01), maximum dimension (R = 0.17; *p* < 0.01), and prolactin (R = 0.16; *p* < 0.01) were all significantly correlated with the Ki-67 index level.

**Conclusions:**

Age, primary-recurrence subtype, maximum dimension, and prolactin are independent predictors for the Ki-67 index level. The current study provides a novel and feasible nomogram, which can further assist neurosurgeons to develop better, more individualized treatment strategies for patients with PAs by predicting the Ki-67 index level preoperatively.

## Introduction

Pituitary adenomas (PAs), which account for approximately 15% of primary intracranial neoplasms, are the most common tumors in the sellar region (1). PAs were recently renamed pituitary neuroendocrine tumors (PitNET), because although they are mostly benign, PAs could present invasive, aggressive, and metastatic behaviors (2). The Ki-67 labeling index is a proliferative marker for PAs (3, 4). A Ki-67 ≥3% is an indicator for invasive growth according to the World Health Organization 2004 classification (5). The French five-tiered classification is another approach for classifying PAs with validated prognostic value, and Ki-67 is used as one of the proliferative markers (2).

The preoperative prediction for the Ki-67 index is valuable and may affect the patient’s surgical approach and postoperative management. A high Ki-67 index requires neurosurgeons to obtain gross-total resection and prepare for higher probability of compression and tissue adhesion in the surrounding structures (6). Furthermore, a high Ki-67 index may prompt a closer follow-up or early radiation therapy (7). Ugga et al. proposed a machine learning model based on T2W magnetic resonance imaging (MRI) for the prediction of the Ki-67 proliferation index class (8). However, how to integrate machine learning model into the routine practices have not been assessed so far (9). Obviously, applying this machine learning model in an actual clinical practice requires supporting software and platform, which must be developed in the future. Conficoni et al. suggested that quantitative measures of apparent diffusion coefficient (ADC) values could predict the Ki67 value (10). However, this value was not routinely assessed in clinical practice.

A nomogram, which is an easy-to-use and graphical predictive tool, has been widely applied to predict numerous binary and prognostic outcomes (11). However, a nomogram for preoperatively forecasting the Ki-67 level in patients with PAs remains unavailable. Therefore, in this retrospective study, we aimed to develop and validate the first predictive nomogram for preoperatively predicting Ki-67 levels in patients with PAs.

## Methods

### Study Design and Patients

We reviewed the clinical records of patients with PAs who underwent PA resection at the Department of Neurosurgery in Jinling Hospital between January 2018 and October 2020. The inclusion criteria were as follows (1): histopathologically confirmed PA (2); patients who underwent PA resection *via* transcranial or transsphenoidal approaches using microscope or endoscope (3); patients with a Ki-67 index identified from histopathological examination results; and (4) patients who had at least one collected variable. The exclusion criteria included the following (1): patients who had no histopathological examination; and (2) patients who had no collected variables. Our institutional research ethics board approved this retrospective study (2016NZKY-008-02). Because of the nature of the retrospective cohort study and the anonymization of data prior to analysis, informed consent was waived.

### Data Collection

Overall, there were 93 variables collected in this study. Baseline characteristics included age, gender, primary-recurrence subtype, treatment history for PAs (e.g., medication, surgery, and radiotherapy), and preoperative signs and symptoms (e.g., moon face, acromegalia, headache, visual impairment, and visual field defect). Radiological features that were collected included maximum dimension of tumor, Knosp grade, Hardy grade, multiple lesions, optic nerve compression, and pituitary apoplexy. Grades 0 to 2 and grades 3 to 4 were classified into noninvasive and invasive classes, respectively, for Knosp grade (12) and Hardy grade for sellar invasion (13). We also collected 74 preoperative laboratory tests, including pituitary hormones, routine blood, coagulation, renal and hepatic functions, and electrolytes, which were based on peripheral blood samples (**Table S2**). Included samples were also diagnosed with clinical subtypes including nonfunctioning, growth hormone (GH) secreting (14, 15), prolactin (PRL) secreting (15), and adrenocorticotropic hormone (ACTH) secreting (16) PAs. Ki-67 index was extracted as postoperatively assessed outcome from the histopathological examination and classified into a low Ki-67 index (<3%) and a high Ki-67 index (≥3%).

### Development and Validation of the Nomogram

First, the patients were randomly divided into a training cohort and a validation cohort. Second, least absolute shrinkage and selection operator (LASSO) algorithm was applied to filter features with a missing data percentage of <60% using glmnet R package (version 4.1). During the LASSO analysis, mean imputation for missing data was applied. Missing data were not imputed in the following analysis to simulate the model performance in real-world conditions. Uni- and multivariate logistic regressions were utilized to determine independent risk factors associated with the Ki-67 level by use of an rms R package (version 6.1.0). In this step, variables have to meet at least one of the following criteria to be included in multivariate logistic regression analysis: displaying a significant difference between the low and high Ki-67 index groups; avoiding being filtered out in the LASSO analysis; or being shown to be a significant predictor in univariate logistic regression analysis. Finally, a nomogram was constructed to visualize the risk factors. The predictions for the validation cohort were calculated using rms R package. The receiver operation characteristic (ROC) curve and the calibration curve were computed separately using pROC (version 1.17.0.1) and rms R packages to evaluate the predictive performance of the nomogram model. We assessed the model performance in the subgroups according to age, gender, primary-recurrence subtype, maximum dimension, clinical subtype, Knosp and Hardy grades, and variables included in the final nomogram. The cutoff values of the continuous variables in the subgroup analysis were the mean values in the validation cohort.

### Sample Size

There were no generally accepted approaches for sample size estimation in the development and validation studies of risk prediction models. However, based on the events per variable (EVP) = 10 criteria (17), the event number in the training cohort should exceed 10 the number of variables included in the multivariate logistic regression analysis. After the filtering process, although there were 21 variables included in the following analysis, we only analyzed all possible combinations up to seven variables in the multivariate logistic regression analysis. Because the current study included 128 events in the training cohort, the sample size was sufficient for analysis in this research.

### Statistical Analysis

Model development and validation were performed according to “Transparent Reporting of a Multivariable Prediction Model for Individual Prognosis or Diagnosis” (TRIPOD) guidance (**Table S1**) (18). The R software (version 3.6.0) was applied for statistical analysis, and statistical significance was set at *p* < 0.05. Continuous variables were presented as the mean ± standard deviation (SD). Comparisons between two continuous variables were evaluated using Student’s *t*-test. The chi-squared test or Fisher’s exact test was used for comparisons of categorized variables. Spearman correlation analysis was applied to evaluate two continuous variables, and the data were visualized using “ggplot” (version 3.3.3) R packages.

## Results

### Baseline Clinical Characteristics of Participants

A total of 439 eligible patients, who were included in the study, were randomly divided into a training cohort (n = 300) and a validation cohort (n = 139). Detailed characteristics for these two cohorts showed homogeneity in these cohorts (**Table S2**). The clinical information of patients with PAs in the high Ki-67 cohort and in the low Ki-67 cohort, which was summarized in **Table 1** and **Table S3**, revealed remarkable differences in the following variables: age, clinical subtype, Hardy grade for suprasellar extension, history of pituitary surgery, multiple lesions, maximum dimension, luteinizing hormone (LH), follicle-stimulating hormone (FSH), free triiodothyronine (FT3), C-reactive protein, red blood cell (RBC) count, creatinine, potassium, procalcitonin (PCT), lymphocyte percentage, urea, mean corpuscular volume (MCV), mean corpuscular hemoglobin (MCH), fibrin/fibrinogen degradation products, thrombocytocrit, and platelet counts.

**Table 1:** Important characteristics of patients with PAs in the low and high Ki-67 cohorts.

Characteristics	Low Ki-67	High Ki-67	*p*
Age (year)	53.48 ± 11.99	46.93 ± 13.69	<0.001*
Gender			0.687
Female	133 (52.2%)	89 (49.7%)	
Male	122 (47.8%)	90 (50.3%)	
Clinical subtype			0.003*
ACTH secreting	7 (2.8%)	4 (2.2%)	
GH secreting	55 (22.2%)	39 (21.8%)	
Nonfunctioning	170 (68.5%)	104 (58.1%)	
PRL secreting	16 (6.5%)	32 (17.9%)	
Primary-recurrence subtype			0.170
Primary	218 (87.9%)	143 (82.7%)	
Recurrence	30 (12.1%)	30 (17.3%)	
Maximum dimension (mm)	26.23 ± 11.12	29.57 ± 12.57	0.010*
Knosp grade			0.706
Noninvasive	123 (57.5%)	74 (54.8%)	
Invasive	91 (42.5%)	61 (45.2%)	
Hardy grade for suprasellar extension			0.009*
0	61 (28.5%)	23 (17.2%)	
A	37 (17.3%)	30 (22.4%)	
B	62 (29%)	36 (26.9%)	
C	47 (22%)	28 (20.9%)	
D	5 (2.3%)	8 (6%)	
E	2 (0.9%)	9 (6.7%)	
Hardy grade for sellar invasion			0.696
Noninvasive	154 (72%)	93 (69.4%)	
Invasive	60 (28%)	41 (30.6%)	
Multiple lesions			0.033*
No	213 (99.5%)	129 (96.3%)	
Yes	1 (0.5%)	5 (3.7%)	
History of pituitary surgery			0.046*
No	223 (87.5%)	143 (79.9%)	
Yes	32 (12.5%)	36 (20.1%)	
Prolacin (mIU/L)	551.82 ± 796.05	909.36 ± 1190.74	0.001*
LH (IU/L)	6.63 ± 8.31	4.09 ± 5.19	0.001*
FSH (IU/L)	17.41 ± 20.23	10.20 ± 11.95	<0.001*
T3 (nmol/L)	1.25 ± 0.34	1.33 ± 0.40	0.058
FT3 (pmol/L)	4.24 ± 0.74	4.49 ± 1.06	0.046*
RBC count (10^12^/L)	4.32 ± 0.50	4.42 ± 0.49	0.038*
MCV (fL)	90.38 ± 4.36	88.55 ± 6.76	0.009*
MCH (pg)	30.34 ± 1.72	29.57 ± 2.57	0.004*
Lymphocyte percentage (%)	34.85 ± 9.01	36.61 ± 8.80	0.046*
Platelet count (10^9^/L)	197.93 ± 58.29	210.79 ± 56.78	0.017*
Thrombocytocrit (%)	0.21 ± 0.06	0.22 ± 0.06	0.022*
FDP (μg/ml)	2.31 ± 2.08	2.02 ± 1.82	0.040*
Potassium (mmol/L)	4.06 ± 0.37	4.13 ± 0.35	0.045*
PCT (μg/L)	0.05 ± 0.04	0.04 ± 0.02	0.011*
CRP (mg/L)	3.04 ± 9.14	1.76 ± 4.29	0.049*
Creatinine (μmol/L)	62.63 ± 18.48	59.46 ± 16.35	0.024*
Urea (mmol/L)	5.34 ± 1.48	4.95 ± 1.34	0.004*

ACTH secreting, adrenocorticotropic hormone secreting; GH secreting, growth hormone secreting; PRL secreting, prolactin secreting; LH, luteinizing hormone; FSH, follicle-stimulating hormone; T3, triiodothyronine; FT3, free triiodothyronine; RBC, red blood cell; MCV, mean corpuscular volume; MCH, mean corpuscular hemoglobin; FDP, fibrin/fibrinogen degradation products; PCT, procalcitonin; CRP, C-reactive protein.

*Statistical significance.

### Filtering Process for Collected Variables

**Figure 1:**
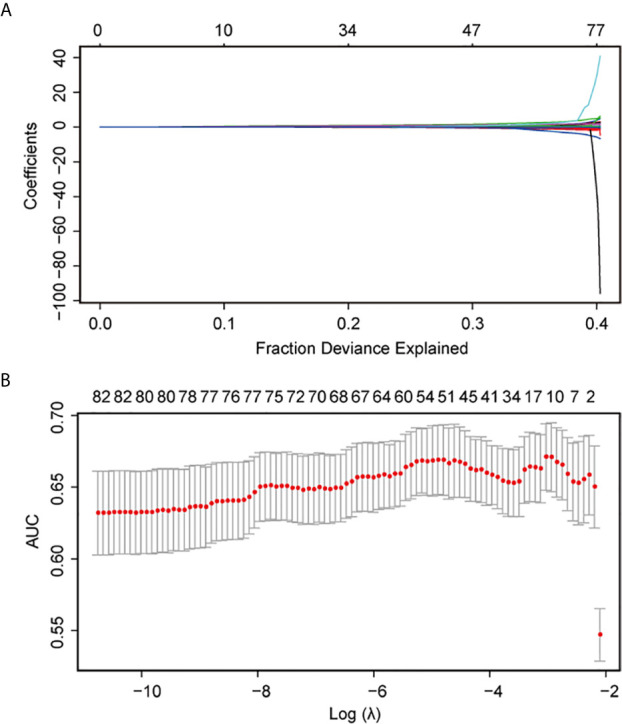
LASSO regression analysis using 10-fold cross-validation. AUC, area under the curve.

Age, primary-recurrence subtype, history of pituitary surgery, clinical subtype, Hardy grade for suprasellar extension, maximum dimension, prolactin, LH, FSH, T3, FT3, RBC count, potassium, PCT, MCV, MCH, thrombocytocrit, and platelet counts were obtained as the primary predictive factors with *p* < 0.05 in a univariate logistic regression analysis (**Table S4**). The results showed that age, LH, FSH, PCT, MCV, and MCH were protective factors for high Ki-67 level, whereas the other primary predictive factors were risk factors. As shown in **Figures 1A** and **B**, a 10-fold cross validation was performed. The following 10 features were screened out from 93 features according to the LASSO analysis (**Table S5**): age, primary-recurrence subtype, clinical subtype, Hardy grade for suprasellar extension, maximum dimension, LH, FSH, FT3, potassium, and MCH.

### Determination of Independent Predictors for the Ki-67 Level

The variables filtered using univariate logistic regression analysis and LASSO analysis were combined and further analyzed using multivariable logistic regression analysis. Finally, as shown in **Table 2**, age (odds ratio [OR], 95% confidence interval [CI]: 0.9687, 0.9473*–*0.9906, *p* = 0.0052), primary-recurrence subtype (OR, 95% CI: 2.2401, 1.0435*–*4.8091, *p* = 0.0385), maximum dimension (OR, 95% CI: 1.0321, 1.0063*–*1.0585, *p* = 0.0144), and prolactin (OR, 95% CI: 1.0004, 1.0000*–*1.0008, *p* = 0.0287) were incorporated into the multivariate model.

**Table 2:** Univariate and multivariate logistic regression analysis for the final model.

Characteristics	Univariate analysis					Multivariate analysis			
	Coefficient	OR	95% CI	*p*		Coefficient	OR	95% CI	*p*
Age (year)	–0.0401	0.9607	0.9426–0.9792	<0.0001*		–0.0318	0.9687	0.9473–0.9906	0.0052*
Primary-recurrence subtype									
Primary	Reference					Reference			
Recurrence	0.7671	2.1536	1.1350–4.0861	0.0189*		0.8065	2.2401	1.0435–4.8091	0.0385*
Maximum dimension (mm)	0.0358	1.0364	1.0132–1.0602	0.0020*		0.0316	1.0321	1.0063–1.0585	0.0144*
Prolacin (mIU/L)	0.0005	1.0005	1.0002–1.0005	0.0027*		0.0004	1.0004	1.0000–1.0008	0.0287*

CI, confidence interval; OR, odds ratio. *Statistical significance.

### Construction and Validation of Nomogram Prediction Model

**Figure 2:**
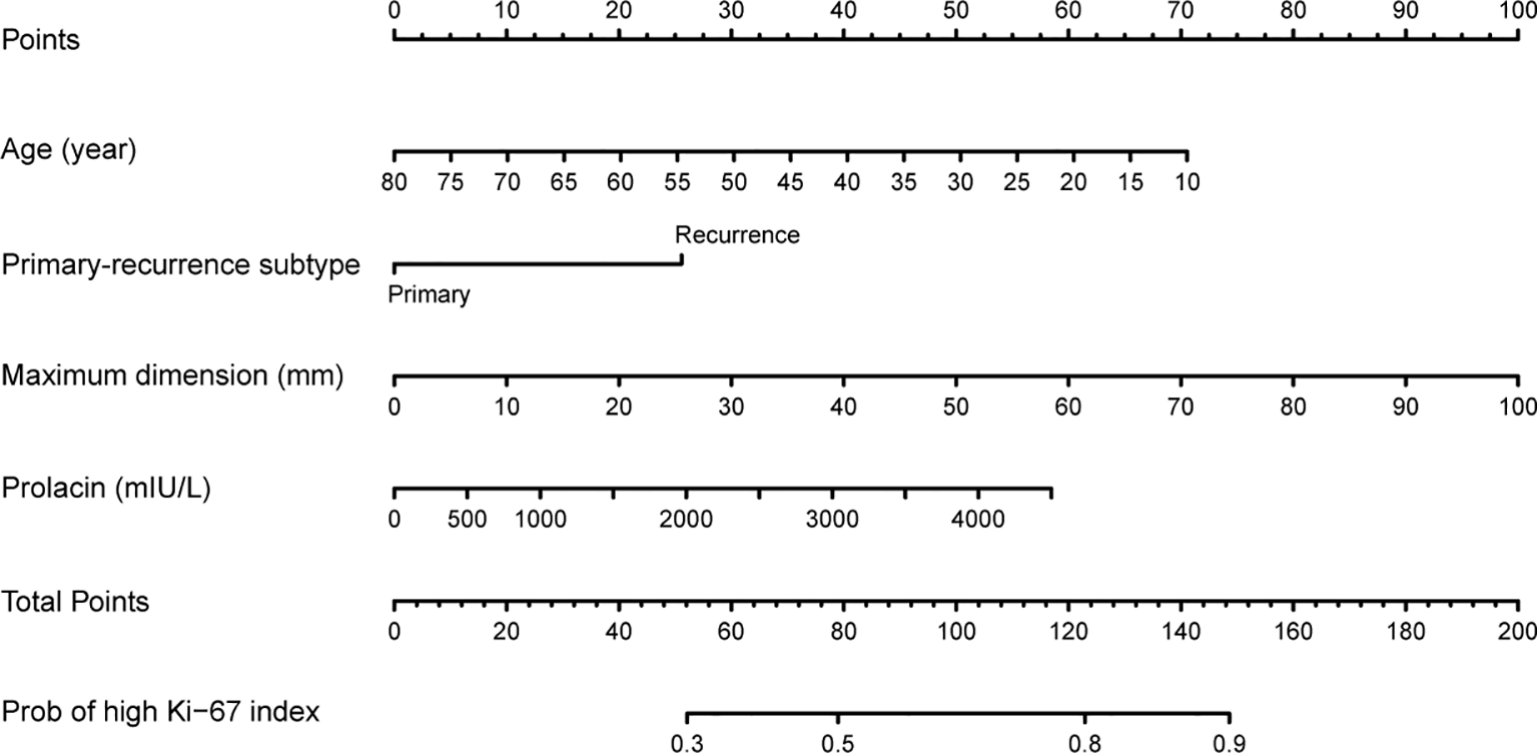
Nomogram for preoperatively predicting the proportion of high Ki-67 index levels for patients with pituitary tumor.

**Figure 3:**
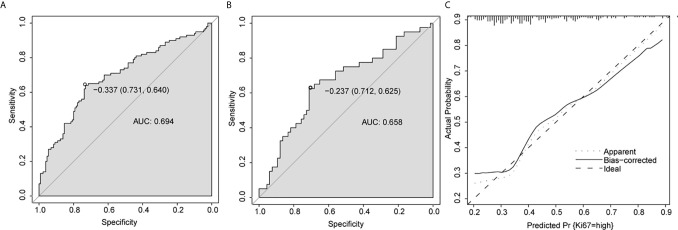
Predictive performance for nomogram. **(A, B)** ROC analysis of nomogram in training cohort **(A)** and validation cohort **(B)**. **(C)** Calibration plots of the nomogram. AUC, area under the curve.

The multivariate model was visualized as a nomogram (**Figure 2**). To apply the nomogram, users should draw a virtual vertical line from each variable to the “Points” axis to identify the points attributed by each variable. Then, users need to compare the summed points with the bottom scale to assess the probability of a high Ki-67 index. The areas under the curve (AUCs) of the training and validation cohorts were 0.694 and 0.658, respectively (**Figures 3A, B** **)**. A well-fitted calibration curve was also generated, which reflected adequate prediction accuracy using the nomogram model (**Figure 3C**). In the subgroup analysis, the nomogram model obtained stable predictive performance in the following subgroups: age (AUC = 0.574 and 0.722 in subgroups >51 years and ≤51 years, respectively), gender (AUC = 0.610 and 0.709 in male and female subgroups, respectively), primary-recurrence subtype (AUC = 0.706 and 0.533 in primary and recurrence subgroups, respectively), maximum dimension (AUC = 0.500, 0.664, and 0.567 in <10, 10*–*40, and ≥40 mm subgroups, respectively), clinical subtype (AUC = 0.635, 0.667, 0.556, and 0.667 in nonfunctioning PAs, GH secreting PAs, PRL secreting PAs, and ACTH secreting PAs subgroups, respectively), Knosp grade (AUC = 0.692 and 0.679 in grades 0*–*2 and grades 3*–*4 subgroups, respectively), Hardy grade (AUC = 0.679 and 0.713 in grades 0*–*2 and grades 3*–*4 subgroups, respectively) and prolactin (AUC = 0.667 and 0.590 in >815 mIU/L and ≤815 mIU/L subgroups, respectively) (**Figure S1** and **Table 3**).

**Table 3:** ROC analysis in subgroups.

Subgroups	AUC
Age (year)	
>51 years	0.574
≤51 years	0.722
Gender	
Female	0.709
Male	0.610
Clinical subtype	
Nonfunctioning	0.635
PRL secreting	0.556
GH secreting	0.667
ACTH secreting	0.667
Primary-recurrence subtype	
Primary	0.706
Recurrence	0.533
Maximum dimension (mm)	
Microadenoma (<10 mm)	0.500
Macroadenoma (10–40 mm)	0.664
Giant adenoma (≥40 mm)	0.567
Knosp grade	
Noninvasive	0.692
Invasive	0.679
Hardy grade for sellar invasion	
Noninvasive	0.679
Invasive	0.713
Prolacin (mIU/L)	
>815 mIU/L	0.667
≤815 mIU/L	0.590

AUC, area under curve.

### Correlation Analysis

We further investigated the relationship between independent predictors and the Ki-67 index. The correlation analysis found that age (R = −0.23; *p* < 0.01), maximum dimension (R = 0.17; *p* < 0.01), and prolactin (R = 0.16; *p* < 0.01) were all significantly correlated with the Ki-67 index (**Figures S2A–C** and **Table 4**). Furthermore, the subgroup analysis revealed that the Ki-67 index was inversely correlated with age in nonfunctioning (R = −0.19; *p* < 0.01) and GH secreting (R = −0.31; *p* < 0.01) PA (**Figures S2D, F** and **Table 4**). However, no significant correlation was found in PRL and ACTH secreting PA (**Figures S2E, G** and **Table 4**). Interestingly, the subgroup analysis according to maximum dimension found a negative correlation between Ki-67 index and a maximum dimension in microadenoma (R = −0.62; *p* = 0.02) (**Figure S2H** and **Table 4**), and positive correlation in macroadenoma (R = 0.17; *p* < 0.01) (**Figure S2I** and **Table 4**). No significant correlation between the Ki-67 index and maximum dimension was found in giant adenoma (**Figure S2J** and **Table 4**). In the subgroup analysis according to clinical types, we discovered a significant positive correlation between Ki-67 index and maximum dimension in GH (R = 0.30; *p* = 0.01) (**Figure S2M** and **Table 4**) and PRL (R = 0.32; *p* = 0.05) (**Figure S2L** and **Table 4**) secreting PA, whereas no significant correlation was found for nonfunctioning and ACTH secreting PA (**Figures S2K, N** and **Table 4**). We conducted a subgroup analysis according to prolactinoma or no prolactinoma, and found that in the no prolactinoma subgroup, the Ki-67 index was significantly correlated with prolactin (R = 0.12; *p* = 0.02) (**Figure S2O** and **Table 4**). However, significant correlation between the Ki-67 index and prolactin was not detected in the prolactinoma subgroup (R = 0.07; *p* = 0.65) (**Figure S2P** and **Table 4**).

**Table 4:** Subgroup analysis of correlation between the Ki-67 index and independent risk factors.

Risk Factor	Subgroups	R	*p*
Age		−0.23	<0.01*
	Nonfunctioning	−0.19	<0.01*
	PRL secreting	−0.14	0.38
	GH secreting	−0.31	<0.01*
	ACTH secreting	0.28	0.44
Maximum dimension		0.17	<0.01*
	Microadenoma (<10 mm)	−0.62	0.02*
	Macroadenoma (10*–*40 mm)	0.17	<0.01*
	Giant adenoma (≥40 mm)	−0.05	0.75
	Nonfunctioning	0.04	0.59
	PRL secreting	0.32	0.05*
	GH secreting	0.30	0.01*
	ACTH secreting	0.25	0.51
Prolacin		0.16	0.01*
	Prolactinoma	0.07	0.65
	No prolactinoma	0.12	0.02*

PRL secreting, prolactin secreting; GH secreting, growth hormone secreting; ACTH secreting, adrenocorticotropic hormone secreting. *Statistical significance.

## Discussion

PA is the most common tumor in the sellar region (1), and high Ki-67 suggests invasive growth and worse prognosis for patients with PAs (2, 5). Preoperative prediction of Ki-67 could further assist neurosurgeons to develop better, more individualized treatment strategies for patients with PAs. However, there was no user-friendly tool for preoperatively predicting Ki-67. In this study, age, primary-recurrence subtype, maximum dimension, and prolactin were identified as independent risk factors for the Ki-67 level. Subsequently, these four predictors were incorporated into a predictive nomogram to preoperatively calculate the risk probability of a high Ki-67 level tailored to individual patients.

To the best of our knowledge, Ugga et al. and Conficoni et al. both proposed methods predicting the Ki-67 index for patients with PAs based on MRI and diffusion-weighted imaging (8, 10). The machine learning model built by Ugga et al. showed high accuracy (91.67%, 33/36). Conficoni et al. suggested that mean apparent diffusion coefficient values had sensitivity and specificity of 90% and 85%, respectively. Although their prediction methods both showed considerable prediction performance, application of these methods was still inconvenient. Our nomogram is the first predictive model for Ki-67 levels based on age, primary-recurrence subtype, maximum dimension, and prolactin. These four parameters were routinely evaluated for patients with PAs in clinical practice, denoting strong practicability for the nomogram. The nomogram showed predictive value (AUC = 0.694 and 0.658 in the training and validation cohorts, respectively) and the performance was stable in various subgroups (**Figure S1** and **Table 3**). Obviously, this user-friendly nomogram was clinically useful and easy to expand in clinical practice.

Yonezawa et al. found that the Ki-67 index in patients under 30 years of age with nonfunctioning PA was significantly higher than that in patients over 40 years of age (*p* < 0.01) (19). Tanaka et al. also discovered that Ki-67 index had an inverse correlation with patient’s age (R = −0.61) in those with nonfunctioning PAs (20). Losa et al. also found similar results that the Ki-67 index was inversely correlated with age at the time of surgery in patients with nonfunctioning PA (21). Recently, Trott et al. detected a significant relationship between Ki-67 and age (*p* = 0.001) in patients with nonfunctioning PA (22). In addition to nonfunctioning PA, Jaffrain-Rea et al. proposed that in patients with clinically secreting PA, Ki-67 index decreased with patient’s age (R = 0.28, *p* = 0.025) (23). In 2019, Mohseni et al. reported that a significantly higher Ki-67 index level (*p* = 0.036) was found in younger patients with GH-secreting PA (24). Conversely, few studies showed no statistical relationship between the Ki-67 index and age (25, 26). However, these opposite results were based on mixed clinical subtypes, which could influence the results as a confounding factor. In the current study, we found that age was significantly higher in the low Ki-67 cohort compared with that in the high Ki-67 cohort (*p* < 0.001) (**Table 1**). A multivariate analysis also suggested that age is an independent predictor for Ki-67 index level (OR, 95% CI: 0.9687, 0.9473*–*0.9906, *p* = 0.0052) (**Table 2**). A correlation analysis revealed a significant inverse correlation between the Ki-67 index and age in all patents with PA (R = −0.23; *p* < 0.01), nonfunctioning PA (R = −0.19; *p* < 0.01), and GH secreting PA (R = −0.31; *p* < 0.01) subgroups (**Figure S2** and **Table 4**). Although a correlation analysis found no statistical significance in PRL and ACTH secreting PA, these results may be due to our relatively small PRL and ACTH secreting PA sample sizes. The predictive performance of the nomogram model was stable across different clinical subgroups, suggesting that this nomogram could be applied to patients with nonfunctioning, GH, PRL, and ACTH secreting PA.

Most of literature discussed whether the Ki-67 index could or could not predict recurrence, but only few studies analyzed the predictive value of primary-recurrence subtype for predicting Ki-67 index levels in PA. Doyle et al. proposed that Ki-67 was raised in recurrent (33%) than primary (11%) PA (*p* < 0.01) (27). Yao et al. found that the mean values of Ki-67 index for nonfunctioning PA were 2.76 and 4.09% for the primary and recurrent groups, respectively (28). Grimm et al. discovered a tendency that Ki-67 index were higher in recurrent ACTH and PRL secreting PA but did not reach statistical significance (29). In contrast, Selek et al. found that among the patients who received somatostatin analogue treatment between the first and second operations, the Ki-67 index was significantly lower at the time of the second operation compared to that of the first operation (*p* < 0.001) (30). However, this decrease in the Ki-67 index may be due to the cellular proliferation depression effect of somatostatin analogues. Based on the uni- and multivariate logistic regression analyses in the current study, the primary-recurrence subtype is an independent predictor for Ki-67 index level (**Table 2**). These results suggested that recurrent PA has a higher probability for a high Ki-67 index compared with primary PA.

The relationship between Ki-67 and maximum dimension remains controversial. Some studies have found no significant correlation between Ki-67 and maximum dimension for mixed clinical subtypes (29, 31, 32), PRL secreting (33), GH secreting (34) and nonfunctioning (35) PAs. In contrast, positive correlations between Ki-67 and maximum dimension in macroadenoma were discovered for mixed clinical subtypes (23, 36) and ACTH secreting (37) PAs. Turner et al. proposed that positive and negative correlations existed between Ki-67 and maximum dimension in macroadenoma and microadenoma, respectively (38). Onishi et al. found a significant difference of Ki-67 index between macroadenoma and microadenoma (macro vs. micro (% ± SEM): 0.62 ± 0.20 vs. 0.04 ± 0.02; *p* = 0.03) in mixed clinical subtypes PA (39). Wierzbicka-Tutka et al. also discovered a higher Ki-67 index in the macroadenoma group than in the microadenoma group (macro vs. micro (median): 1.4% vs. 1.03%; *p* = 0.02) in the mixed clinical subtypes PA (40). Li et al. found similar results: Ki-67 was correlated with maximum dimension (*p* < 0.05) in the mixed clinical subtypes PA (41). Ramírez et al. discovered that in nonfunctioning PA, the Ki-67 index was associated with a maximum dimension >3 cm (OR, 95% CI: 2.32, 1.17–4.58; *p* = 0.01) (42). Baldys-Waligorska et al. revealed that in GH secreting PA, Ki-67 was correlated with maximum dimension (R = 0.42, *p* = 0.025) (43). It seems that maximum dimension subtypes and clinical subtypes should be considered while discussing Ki-67 and maximum dimension for PA. In our study, first, we found that maximum dimension was significantly lower in the low Ki-67 cohort than in the high Ki-67 cohort (low Ki-67 vs. high Ki-67 (mean ± SD): 26.23 ± 11.12 vs. 26.23 ± 11.12; *p* = 0.01) (**Table 1**). The uni- and multivariate analyses revealed that the maximum dimension was an independent risk factor for a high Ki-67 index (OR, 95% CI: 1.0321, 1.0063*–*1.0585, *p* = 0.0144) (**Table 2**). In the correlation analysis, a positive correlation between the Ki-67 index and maximum dimension was found for all of the included patients with PA (R = 0.17; *p* < 0.01) (**Figure S2B** and **Table 4**). In the subgroup, according to the maximum dimension subtype and clinical subtype, we discovered a positive correlation and a negative correlation between the Ki-67 index and maximum dimension in the macroadenoma (R = 0.17; *p* < 0.01) and microadenoma (R = −0.62; *p* = 0.02) subgroups, respectively (**Figures S2H, I** **;** **Table 4**), which was consistent with the results from the research by Turner et al. A significant positive correlation between Ki-67 index and maximum dimension was also revealed in GH (R = 0.30; *p* = 0.01) and PRL (R = 0.32; *p* = 0.05) secreting PA subgroups (**Figures S2L, M** and **Table 4**). These results require further verification and we propose that additional discussion of Ki-67 and maximum dimension for PA should consider maximum dimension subtypes and clinical subtypes before drawing conclusions.

In this study, we found that the preoperative prolactin concentration was significantly lower in the low Ki-67 cohort compared with that in the high Ki-67 cohort (low Ki-67 vs. high Ki-67 (mean ± SD): 551.82 ± 796.05 vs. 909.36 ± 1190.74; *p* = 0.001) (**Table 1**). The prolactin concentration was an independent predictor for the Ki-67 index level (OR, 95% CI: 1.0004, 1.0000*–*1.0008, *p* = 0.0287) (**Table 2**). Few studies have discussed the relationship between Ki-67 index and preoperative serum prolactin concentration. Lu et al. found that the preoperative PRL levels in the Ki-67 index >3% group remained significantly higher compared to those in patients with the Ki-67 index <3% group (*p* < 0.05) (44). Fedorova et al. (45) and Zielinski et al. (46) confirmed a positive correlation between the Ki-67 index and preoperative prolactin concentration in patients with prolactinoma. Li et al. proposed that ethanol altered hypothalamic neurotransmitter levels and stimulated anterior pituitary cell proliferation, which further increased the prolactin level in cells and the secretory output of prolactin (47). This theory may suggest a potential mechanism for the positive correlation between the Ki-67 index and prolactin, but additional high-quality research is warranted for validation. In the current work, we analyzed the relationship between the Ki-67 index and prolactin in the prolactinoma and no prolactinoma subgroups and found a significant correlation in the no prolactinoma subgroup (**Figures S2O, P** and **Table 4**). One possible reason for these interesting results is that in the no prolactinoma subgroup, the positive correlation between the Ki-67 index and prolactin is due to “stalk effect.” Higher Ki-67 tends to show up in larger PAs and leads to a more severe “stalk effect.” Because dopamine inhibits the basally high-secretory tone of lactotrophs, when the tumor mass blocks the infundibular dopamine release, hyperprolactinemia will occur (48).

Knosp classification, based on the relationship between PA and internal carotid artery, is used to evaluate invasion of the cavernous sinus. Luchi et al. found the Knosp grade was significantly correlated with the Ki-67 index (R = 0.73; *p* < 0.001) in GH secreting PA population (49). Recently, Li et al. also revealed that the Knosp grade positively correlated with the Ki-67 index (*p <*0.05) (41). However, another study (50) reported an inverse relationship between the Ki-67 index and Knosp grade for functional PA and nonfunctional PA (R= -0.59, *p* < 0.001 in functional PA and R = 0.367, *p* < 0.01 in nonfunctional PA). Das et al. found no significant correlation between Knosp grade and Ki-67 index (5). In the current research, PAs with the Knosp grades 0*–*2 and grades 3*–*4 were classified into noninvasive and invasive classes. PAs were also classified into a low Ki-67 index (<3%) and high Ki-67 index (≥3%). No significant correlation was found between the Knosp grade and Ki-67 index (**Table 4** and **Supplementary Table 4**). The difference between our results and previous studies’ results may be due to our inclusion criteria, based on which we included both functional and nonfunctional PA patients.

This study had several limitations. First, as a single-institution retrospective study, potential selection bias derived from some unknown factors is inevitable and may decrease the reliability of the results. And because no thyrotropinoma, and gonadotropinoma was treated in our center, the current study only included nonfunctioning, GH secreting, PRL secreting, and ACTH secreting PAs. The lack of thyrotropinoma, and gonadotropinoma may lead to selection bias, which cannot be ignored. Therefore, additional investigations from external sources are warranted to comprehensively evaluate and validate the nomogram. Second, the predictive performance of the nomogram and that in some subgroups were unsatisfactory, e.g., microadenoma. However, this is the first nomogram to preoperatively predict the Ki-67 level for PAs, and the model did show predictive value to some extent. So, we recommended clinicians to apply this nomogram in clinical. But clinicians had to carefully select the appropriate patients while applying this nomogram. For example, patients with following characteristics are more suitable for this nomogram: under 51 years old (AUC = 0.722), female (AUC = 0.709), primary subtype (AUC = 0.706) or invasive with Hardy grade for sellar invasion (AUC = 0.713). In the future, additional nomogram studies are warranted to focus on the appropriate and eligible subgroups of patients. Third, several variables collected in this study had missing data. However, we checked all of the variables filtered by the LASSO and univariate regression analyses, and all of these analyses had missing data under 60%. Furthermore, in this study, the percentage of missing data in the final model was only 23.3%, which is a satisfactory and adequate sample size for a multivariate analysis. Finally, we found a different relationship between the Ki-67 index and its independent risk factors in the subgroup analysis; thus, a better nomogram should be based on some subgroup populations. Because the current work focused on all patients with PAs, we did not calculate nomograms for specific subgroup populations. Additional studies are warranted to analyze the prediction of the Ki-67 index in these subgroup populations.

## Conclusions

This study showed that age, primary-recurrence subtype, maximum dimension, and prolactin are independent risk factors associated with the Ki-67 level. Among these risk factors, prolactin was found for the first time to be an independent predictor for the Ki-67 level. The novel nomogram developed in this study was feasible and stable for preoperative prediction of the Ki-67 index level, suggesting that the nomogram is of potential value for further assisting neurosurgeons to develop better, more individualized treatment strategies for patients with PAs.

## Data Availability Statement

The raw data supporting the conclusions of this article will be made available by the authors, without undue reservation.

## Ethics Statement

The studies involving human participants were reviewed and approved by the Ethics Committee of Jinling Hospital. Written informed consent for participation was not provided by the participants’ legal guardians/next of kin because of the nature of the retrospective cohort study and the anonymization of data prior to analysis, informed consent was waived.

## Author Contributions

CM conceived and designed the investigation. XC analyzed the data and drafted the manuscript. JZ, JY, CT, FY, and ZC conducted statistical analyses. All authors contributed to the article and approved the submitted version.

## Conflict of Interest

The authors declare that the research was conducted in the absence of any commercial or financial relationships that could be construed as a potential conflict of interest.

## Supplementary Material

Supplementary Table 1 TRIPOD checklist for prediction model development and validation.

Supplementary Table 2 Characteristics of patients with PA in the training and validation cohorts.

Supplementary Table 3 Other characteristics of patients with PA in the low and high Ki-67 cohorts.

Supplementary Table 4 Univariate logistic regression analysis of features in the training cohort.

Supplementary Table 5 LASSO analysis in the training cohort.

Supplementary Figure 1 Predictive performance for the nomogram in the subgroup analysis. **(A, B)** ROC analysis in the subgroup according to age (**A** for >51 years and **B** for ≤51 years); **(C, D)** ROC analysis in the subgroup according to gender (**C** for female and **D** for male); **(E*–*H)** ROC analysis in the subgroup according to clinical subtypes (**E** for nonfunctioning; **F** for PRL secreting; **G** for GH secreting; and **H** for ACTH secreting pituitary tumors); **(I, J)** ROC analysis in the subgroup according to primary-recurrence subtypes (**I** for primary and **J** for recurrence subtypes); **(K, L)** ROC analysis in the subgroup according to Knosp grade (**K** for noninvasive and **L** for invasive); **(M, N)** ROC analysis in the subgroup according to Hardy grade (**M** for noninvasive and **N** for invasive); **(O, P)** ROC analysis in the subgroup according to prolactin (**O** for >815 mIU/L and **P** for ≤815 mIU/L); **(Q*–*S)** ROC analysis in the subgroup according to the maximum dimension (**Q** for microadenoma, **R** for macroadenoma, and **S** for giant adenoma).

Supplementary Figure 2 Correlation analysis. **(A*–*C)** Correlation between Ki-67 index and age **(A)**, maximum dimension **(B)**, and prolactin **(C)**, respectively, in all of the validation cohort. **(D*–*G)** Correlation between Ki-67 index and age in the subgroups according to nonfunctioning **(D)**, PRL secreting **(E)**, GH secreting **(F)**, and ACTH secreting **(G)** pituitary tumors. **(H*–*N)** correlation between Ki-67 index and maximum dimension in the subgroups according to microadenoma **(H)**, macroadenoma **(I)**, giant adenoma **(J)**, nonfunctioning **(K)**, PRL secreting **(L)**, GH secreting **(M)**, and ACTH secreting **(N)** pituitary tumors. **(O*–*P)** correlation between Ki-67 index and prolactin in the subgroups according to prolactinoma **(O)** and no prolactinoma **(P)**.

## References

[1] Melmed S. Pituitary-Tumor Endocrinopathies. N Engl J Med (2020) 382:937–50. 10.1056/NEJMra1810772 32130815

[2] Trouillas J Jaffrain-Rea M-L Vasiljevic A Raverot G Roncaroli F Villa C. How to Classify the Pituitary Neuroendocrine Tumors (PitNET)s in 2020. Cancers (2020) 12:514. 10.3390/cancers12020514 PMC7072139 32098443

[3] Asioli S Righi A Iommi M Baldovini C Ambrosi F Guaraldi F. Validation of a Clinicopathological Score for the Prediction of Post-Surgical Evolution of Pituitary Adenoma: Retrospective Analysis on 566 Patients From a Tertiary Care Centre. Eur J Endocrinol (2019) 180:127–34. 10.1530/eje-18-0749 30481158

[4] Kim JS Lee YS Jung MJ Hong YK. The Predictive Value of Pathologic Features in Pituitary Adenoma and Correlation With Pituitary Adenoma Recurrence. J Pathol Transl Med (2016) 50:419–25. 10.4132/jptm.2016.06.30 PMC5122726 27713217

[5] Das C Mondal P Mukhopadhyay M Mukhopadhyay S Ghosh I Handral A. Evaluation of Prognostic Utility of Ki-67, P53, and O-6-methylguanine-DNA Methyltransferase Expression in Pituitary Tumors. J Lab physicians (2019) 11:323–9. 10.4103/JLP.JLP_76_19 PMC6943861 31929698

[6] Dai C Liu X Ma W Wang R. The Treatment of Refractory Pituitary Adenomas. Front Endocrinol (Lausanne) (2019) 10:334. 10.3389/fendo.2019.00334 31191457 PMC6548863

[7] Gerges MM Rumalla K Godil SS Younus I Elshamy W Dobri GA. Long-Term Outcomes After Endoscopic Endonasal Surgery for Nonfunctioning Pituitary Macroadenomas. J Neurosurg (2020) 134:1–12. 10.3171/2019.11.Jns192457 32005016

[8] Ugga L Cuocolo R Solari D Guadagno E D’Amico A Somma T. Prediction of High Proliferative Index in Pituitary Macroadenomas Using MRI-based Radiomics and Machine Learning. Neuroradiology (2019) 61:1365–73. 10.1007/s00234-019-02266-1 31375883

[9] Peiffer-Smadja N Dellière S Rodriguez C Birgand G Lescure FX Fourati S. Machine Learning in the Clinical Microbiology Laboratory: Has the Time Come for Routine Practice? Clin Microbiol Infect (2020) 26:1300–9. 10.1016/j.cmi.2020.02.006 32061795

[10] Conficoni A Feraco P Mazzatenta D Zoli M Asioli S Zenesini C. Biomarkers of Pituitary Macroadenomas Aggressive Behaviour: A Conventional MRI and DWI 3T Study. Br J Radiol (2020) 93:20200321. 10.1259/bjr.20200321 32628097 PMC7465851

[11] Iasonos A Schrag D Raj GV Panageas KS. How to Build and Interpret a Nomogram for Cancer Prognosis. J Clin Oncol (2008) 26:1364–70. 10.1200/JCO.2007.12.9791 18323559

[12] Knosp E Steiner E Kitz K Matula C. Pituitary Adenomas With Invasion of the Cavernous Sinus Space: A Magnetic Resonance Imaging Classification Compared With Surgical Findings. Neurosurgery (1993) 33:610–7; discussion 7-8. 10.1227/00006123-199310000-00008 8232800

[13] Hardy J Somma M. Surgical Treatment by Transsphenoidal Microsurgical Removal of the Pituitary Adenoma. In: Colins W Tindall G, editors. Clinical Management of Pituitary Disorders. New York: Raven (1979). p. 209–17.

[14] de Pablos-Velasco P Venegas EM Álvarez Escolá C Fajardo C de Miguel P González N. Diagnosis, Treatment and Follow-Up of Patients With Acromegaly in a Clinical Practice Setting in Spain: The ACROPRAXIS Program Delphi Survey. Pituitary (2020) 23:129–39. 10.1007/s11102-019-01012-3 PMC7066268 31823249

[15] Gada JV Sanamandra P Barasara SA Chauhan YV Bhagwat NM. Current Status of Diagnosis and Management of Functioning Pituitary Tumors: Part II. Neurol India (2020) 68:S20–s7. 10.4103/0028-3886.287672 32611888

[16] Thakkar K Sarathi V Shah NS. Current Status of Diagnosis and Management for Functioning Pituitary Tumors: Part I. Neurol India (2020) 68:S13–s9. 10.4103/0028-3886.287680 32611887

[17] Peduzzi P Concato J Kemper E Holford TR Feinstein AR. A Simulation Study of the Number of Events Per Variable in Logistic Regression Analysis. J Clin Epidemiol (1996) 49:1373–9. 10.1016/s0895-4356(96)00236-3 8970487

[18] Moons KG Altman DG Reitsma JB Ioannidis JP Macaskill P Steyerberg EW. Transparent Reporting of a Multivariable Prediction Model for Individual Prognosis or Diagnosis (TRIPOD): Explanation and Elaboration. Ann Intern Med (2015) 162:W1–73. 10.7326/m14-0698 25560730

[19] Yonezawa K Tamaki N Kokunai T. Clinical Features and Growth Fractions of Pituitary Adenomas. Surg Neurol (1997) 48:494–500. 10.1016/s0090-3019(97)00102-x 9352815

[20] Tanaka Y Hongo K Tada T Sakai K Kakizawa Y Kobayashi S. Growth Pattern and Rate in Residual Nonfunctioning Pituitary Adenomas: Correlations Among Tumor Volume Doubling Time, Patient Age, and MIB-1 Index. J Neurosurg (2003) 98:359–65. 10.3171/jns.2003.98.2.0359 12593623

[21] Losa M Franzin A Mangili F Terreni MR Barzaghi R Veglia F. Proliferation Index of Nonfunctioning Pituitary Adenomas: Correlations With Clinical Characteristics and Long-Term Follow-Up Results. Neurosurgery (2000) 47:1313–8; discussion 8-9. 10.1097/00006123-200012000-00009 11126902

[22] Trott G Ongaratti BR de Oliveira Silva CB Abech GD Haag T Rech C. PTTG Overexpression in Non-Functioning Pituitary Adenomas: Correlation With Invasiveness, Female Gender and Younger Age. Ann Diagn Pathol (2019) 41:83–9. 10.1016/j.anndiagpath.2019.04.016 31154064

[23] Jaffrain-Rea ML Di Stefano D Minniti G Esposito V Bultrini A Ferretti E. A Critical Reappraisal of MIB-1 Labelling Index Significance in a Large Series of Pituitary Tumours: Secreting Versus Non-Secreting Adenomas. Endocr Relat Cancer (2002) 9:103–13. 10.1677/erc.0.0090103 12121834

[24] Mohseni S Aboeerad M Sharifi F Tavangar SM Mohajeri-Tehrani M. Associations of Ki-67 Labeling Index With Clinical and Paraclinical Features of Growth Hormone-Secreting Pituitary Adenomas: A Single Center Report From Iran. Int J Endocrinol Metab (2019) 17:e81983. 10.5812/ijem.81983 31372169 PMC6628618

[25] Mastronardi L Guiducci A Puzzilli F. Lack of Correlation Between Ki-67 Labelling Index and Tumor Size of Anterior Pituitary Adenomas. BMC Cancer (2001) 1:12. 10.1186/1471-2407-1-12 11570981 PMC56633

[26] Pizarro CB Oliveira MC Coutinho LB Ferreira NP. Measurement of Ki-67 Antigen in 159 Pituitary Adenomas Using the MIB-1 Monoclonal Antibody. Braz J Med Biol Res (2004) 37:235–43. 10.1590/s0100-879x2004000200011 14762579

[27] Doyle PM Thiryayi WA Joshi A du Plessis D Kearney T Gnanalingham KK. Beta Human Chorionic Gonadotropin (beta-hCG) Expression in Pituitary Adenomas: Relationship to Endocrine Function and Tumour Recurrence. Pituitary (2009) 12:190–5. 10.1007/s11102-008-0155-x 19005764

[28] Yao X Gao H Li C Wu L Bai J Wang J. Analysis of Ki67, Hmga1, MDM2, and RB Expression in Nonfunctioning Pituitary Adenomas. J Neurooncol (2017) 132:199–206. 10.1007/s11060-016-2365-9 28255749 PMC5378727

[29] Grimm F Maurus R Beschorner R Naros G Stanojevic M Gugel I. Ki-67 Labeling Index and Expression of p53 Are Non-Predictive for Invasiveness and Tumor Size in Functional and Nonfunctional Pituitary Adenomas. Acta Neurochir (Wien) (2019) 161:1149–56. 10.1007/s00701-019-03879-4 31037500

[30] Selek A Cetinarslan B Canturk Z Tarkun I Hanazay Y Vural C. The Effect of Somatostatin Analogues on Ki-67 Levels in GH-secreting Adenomas. Growth Horm IGF Res (2019) 45:1–5. 10.1016/j.ghir.2019.01.001 30731342

[31] Gejman R Swearingen B Hedley-Whyte ET. Role of Ki-67 Proliferation Index and p53 Expression in Predicting Progression of Pituitary Adenomas. Hum Pathol (2008) 39:758–66. 10.1016/j.humpath.2007.10.004 18439942

[32] Sarkar S Chacko AG Chacko G. An Analysis of Granulation Patterns, MIB-1 Proliferation Indices and p53 Expression in 101 Patients With Acromegaly. Acta Neurochir (Wien) (2014) 156:2221–30; discussion 30. 10.1007/s00701-014-2230-6 25238988

[33] Delgrange E Trouillas J Maiter D Donckier J Tourniaire J. Sex-Related Difference in the Growth of Prolactinomas: A Clinical and Proliferation Marker Study. J Clin Endocrinol Metab (1997) 82:2102–7. 10.1210/jcem.82.7.4088 9215279

[34] Fusco A Zatelli MC Bianchi A Cimino V Tilaro L Veltri F. Prognostic Significance of the Ki-67 Labeling Index in Growth Hormone-Secreting Pituitary Adenomas. J Clin Endocrinol Metab (2008) 93:2746–50. 10.1210/jc.2008-0126 18460561

[35] Ferreira JE de Mello PA de Magalhães AV Botelho CH Naves LA Nosé V. [Non-Functioning Pituitary Adenomas: Clinical Features and Immunohistochemistry]. Arq Neuropsiquiatr (2005) 63:1070–8. 10.1590/s0004-282x2005000600029 16400431

[36] Hasanov R Aydoğan B Kiremitçi S Erden E Güllü S. The Prognostic Roles of the Ki-67 Proliferation Index, P53 Expression, Mitotic Index, and Radiological Tumor Invasion in Pituitary Adenomas. Endocr Pathol (2019) 30:49–55. 10.1007/s12022-018-9563-2 30610566

[37] Losa M Barzaghi RL Mortini P Franzin A Mangili F Terreni MR. Determination of the Proliferation and Apoptotic Index in Adrenocorticotropin-Secreting Pituitary Tumors: Comparison Between Micro- and Macroadenomas. Am J Pathol (2000) 156:245–51. 10.1016/s0002-9440(10)64725-6 PMC1868637 10623673

[38] Turner HE Nagy Z Gatter KC Esiri MM Wass JA Harris AL. Proliferation, Bcl-2 Expression and Angiogenesis in Pituitary Adenomas: Relationship to Tumour Behaviour. Br J Cancer (2000) 82:1441–5. 10.1054/bjoc.1999.1074 PMC2363363 10780524

[39] Onishi K Kamida T Momii Y Abe T Fujiki M. The Clinical and Pathological Significance of Nitric Oxide Synthase in Human Pituitary Adenomas: A Comparison With MIB-1. Endocrine (2014) 46:154–9. 10.1007/s12020-013-0046-4 24008756

[40] Wierzbicka-Tutka I Sokołowski G Bałdys-Waligórska A Adamek D Radwańska E Gołkowski F. PTTG and Ki-67 Expression in Pituitary Adenomas. Przegl Lek (2016) 73:53–8. 27197423

[41] Li C Wei L Li L Wang J Li R Zhang Q. Predicting Short-Term Recurrence in Pituitary Adenomas: Phosphohistone-H3 (Ser 10) Proves an Effective Biomarker. Clin Neuropathol (2020) 39:70–9. 10.5414/np301212 31724532

[42] Ramírez C Cheng S Vargas G Asa SL Ezzat S González B. Expression of Ki-67, Pttg1, FGFR4, and SSTR 2, 3, and 5 in Nonfunctioning Pituitary Adenomas: A High Throughput TMA, Immunohistochemical Study. J Clin Endocrinol Metab (2012) 97:1745–51. 10.1210/jc.2011-3163 22419713

[43] Baldys-Waligorska A Wierzbicka I Sokolowski G Adamek D Golkowski F. Markers of Proliferation and Invasiveness in Somatotropinomas. Endokrynol Pol (2018) 69:182–9. 10.5603/EP.a2018.0001 29334118

[44] Lu C Ren Z Huan C Cui G. The Role of Ki-67 in Women With a Resistant Prolactinoma: A Retrospective Analysis in 199 Hospitalized Patients Over a Period of 5 Years. Pak J Pharm Sci (2014) 27:1075–81. 25016269

[45] Fedorova NS Abrosimov AY Dzeranova LK Pigarova EA Dedov II. [Pituitary Lactotroph Adenomas Resistant to Dopamine Agonist Treatment: Histological and Immunohistochemical Characteristics]. Arkh Patol (2018) 80:34–9. 10.17116/patol201880334-39 29927438

[46] Zielinski G Ozdarski M Maksymowicz M Szamotulska K Witek P. Prolactinomas: Prognostic Factors of Early Remission After Transsphenoidal Surgery. Front Endocrinol (Lausanne) (2020) 11:439. 10.3389/fendo.2020.00439 32733387 PMC7358351

[47] Li N Shi X Fu S Zhu F Yang S. Chronic Alcohol Administration Increases Serum Prolactin Level and Pituitary Cell Proliferation, and Alters Hypothalamus Neurotransmitters in Rat. Neuro Endocrinol Lett (2011) 32:170–5. 21552201

[48] Schwetye KE Dahiya SM. Sellar Tumors. Surg Pathol Clin (2020) 13:305–29. 10.1016/j.path.2020.02.006 32389269

[49] Iuchi S Saeki N Uchino Y Higuchi Y Tatsuno I Nakamura S. Cavernous Sinus Invasion and Tumor Proliferative Potential of Growth Hormone-Producing Pituitary Tumors. Endocr J (2000) 47 Suppl:S77–9. 10.1507/endocrj.47.supplmarch_s77 10890190

[50] Yuhan L Zhiqun W Jihui T Renlong P. Ki-67 Labeling Index and Knosp Classification of Pituitary Adenomas. Br J Neurosurg (2021). 10.1080/02688697.2021.1884186 33905276

