# Supplementary material for: A Nomogram for Preoperatively Predicting the Ki-67 Index of a Pituitary Tumor: A Retrospective Cohort Study
Source: Front Oncol. 2021 May 31;11:687333. doi: 10.3389/fonc.2021.687333 (PMC8200848; doi:10.3389/fonc.2021.687333)
Supplement: Supplementary Table 2 — Characteristics of patients with PA in the training and validation cohorts. [file Table_2.docx]

Supplementary Table 2. Characteristics of patients with PA in the training and validation cohorts

| Characteristics | Training cohort | Validation cohort | *p* |
| --- | --- | --- | --- |
| Ki-67 |  |  | 0.294 |
| Low | 169 (56.9%) | 86 (62.8%) |  |
| High | 128 (43.1%) | 51 (37.2%) |  |
| Age (year) | 50.64±13.01 | 51.04±13.19 | 0.757 |
| Gender |  |  | 0.546 |
| Female | 151 (50.3%) | 75 (54%) |  |
| Male | 149 (49.7%) | 64 (46%) |  |
| Clinical subtype |  |  | 0.056 |
| ACTH secreting | 4 (1.4%) | 7 (5.1%) |  |
| GH secreting | 71 (24%) | 24 (17.6%) |  |
| Nonfunctioning | 191 (64.5%) | 87 (64%) |  |
| PRL secreting | 30 (10.1%) | 18 (13.2%) |  |
| Primary-recurrence subtype |  |  | 0.175 |
| Primary | 246 (84%) | 119 (89.5%) |  |
| Recurrence | 47 (16%) | 14 (10.5%) |  |
| Maximum dimension (mm) | 28.15±12.10 | 26.21±11.32 | 0.177 |
| Knosp grade |  |  | 0.552 |
| Noninvasive | 133 (55.4%) | 66 (59.5%) |  |
| Invasive | 107 (44.6%) | 45 (40.5%) |  |
| Hardy grade for suprasellar extension |  |  | 0.471 |
| 0 | 58 (24.3%) | 28 (25.2%) |  |
| A | 48 (20.1%) | 19 (17.1%) |  |
| B | 68 (28.5%) | 30 (27%) |  |
| C | 46 (19.2%) | 29 (26.1%) |  |
| D | 9 (3.8%) | 4 (3.6%) |  |
| E | 10 (4.2%) | 1 (0.9%) |  |
| Hardy grade for sellar invasion |  |  | 0.710 |
| Noninvasive | 172 (72%) | 77 (69.4%) |  |
| Invasive | 67 (28%) | 34 (30.6%) |  |
| Multiple lesions |  |  | 0.182 |
| No | 233 (97.5%) | 111 (100%) |  |
| Yes | 6 (2.5%) | 0 (0%) |  |
| Optic nerve compression |  |  | 1.000 |
| No | 81 (33.9%) | 38 (34.2%) |  |
| Yes | 158 (66.1%) | 73 (65.8%) |  |
| Pituitary apoplexy |  |  | 0.096 |
| No | 178 (74.2%) | 72 (64.9%) |  |
| Yes | 62 (25.8%) | 39 (35.1%) |  |
| Headache |  |  | 0.583 |
| No | 229 (76.3%) | 102 (73.4%) |  |
| Yes | 71 (23.7%) | 37 (26.6%) |  |
| Visual impairment |  |  | 1.000 |
| No | 143 (47.8%) | 66 (47.5%) |  |
| Yes | 156 (52.2%) | 73 (52.5%) |  |
| Visual field defect |  |  | 0.400 |
| No | 215 (71.9%) | 106 (76.3%) |  |
| Yes | 84 (28.1%) | 33 (23.7%) |  |
| Moon face |  |  | 0.014* |
| No | 297 (99%) | 132 (95%) |  |
| Yes | 3 (1%) | 7 (5%) |  |
| Acromegalia |  |  | 0.038* |
| No | 245 (81.7%) | 125 (89.9%) |  |
| Yes | 55 (18.3%) | 14 (10.1%) |  |
| History of pituitary surgery |  |  | 0.345 |
| No | 249 (83%) | 121 (87.1%) |  |
| Yes | 51 (17%) | 18 (12.9%) |  |
| History of medication |  |  | 0.943 |
| No | 285 (95%) | 133 (95.7%) |  |
| Yes | 15 (5%) | 6 (4.3%) |  |
| History of radiotherapy |  |  | 0.684 |
| No | 296 (98.7%) | 136 (97.8%) |  |
| Yes | 4 (1.3%) | 3 (2.2%) |  |
| Prolacin (mIU/L) | 644.29±925.84 | 815.35±1113.09 | 0.124 |
| Testosterone (nmol/L) | 4.33±5.12 | 4.58±5.22 | 0.648 |
| Estradiol (pmol/L) | 161.44±231.94 | 181.15±217.27 | 0.111 |
| Progesterone (nmol/L) | 2.64±6.24 | 2.67±6.79 | 0.953 |
| LH (IU/L) | 5.94±8.04 | 5.08±6.41 | 0.411 |
| FSH (IU/L) | 15.15±19.60 | 13.36±14.16 | 0.989 |
| DHEAS (umol/L) | 3.78±2.88 | 3.76±2.80 | 0.985 |
| TSH (mIU/L) | 2.25±3.01 | 2.06±1.54 | 0.728 |
| T3 (nmol/L) | 1.27±0.34 | 1.30±0.43 | 0.775 |
| T4 (nmol/L) | 97.42±26.84 | 99.13±25.83 | 0.544 |
| FT3 (pmol/L) | 4.36±0.90 | 4.32±0.87 | 0.333 |
| FT4 (pmol/L) | 9.52±2.91 | 9.71±2.89 | 0.446 |
| ACTH (pg/ml) | 27.27±18.27 | 31.16±43.22 | 0.827 |
| Cortisol (μmol/L) | 0.28±0.15 | 0.28±0.14 | 0.844 |
| IGF-1 (ng/ml) | 247.90±250.27 | 278.05±304.52 | 0.809 |
| IGFBP3 (mg/L) | 5.23±2.32 | 5.03±2.16 | 0.386 |
| GH (μg/L) | 4.00±9.07 | 2.91±7.79 | 0.859 |
| RBC count (10^12/L) | 4.34±0.51 | 4.41±0.48 | 0.127 |
| HCT (%) | 0.39±0.04 | 0.40±0.04 | 0.103 |
| RDW (%) | 13.11±1.43 | 12.81±0.64 | 0.466 |
| MCV (fL) | 89.53±5.92 | 89.79±4.68 | 0.956 |
| MCH (pg) | 29.93±2.32 | 30.18±1.70 | 0.952 |
| Hemoglobin (g/L) | 129.49±16.15 | 132.88±13.30 | 0.101 |
| MCHC (g/L) | 334.27±13.18 | 336.26±11.61 | 0.195 |
| WBC count (10^9/L) | 5.43±1.47 | 5.67±1.71 | 0.237 |
| Neutrophil percentage (%) | 53.19±9.46 | 55.15±9.97 | 0.056 |
| Lymphocyte percentage (%) | 36.13±8.85 | 34.58±9.17 | 0.101 |
| Monocyte percentage (%) | 7.58±1.89 | 7.38±1.84 | 0.270 |
| Basophil percentage (%) | 0.43±0.27 | 0.40±0.24 | 0.509 |
| Eosinophil percentage (%) | 2.67±2.18 | 2.48±1.58 | 0.904 |
| Platelet count (10^9/L) | 204.94±62.48 | 200.22±46.30 | 0.699 |
| Thrombocytocrit (%) | 0.22±0.06 | 0.21±0.04 | 0.564 |
| MPV (fL) | 10.87±1.35 | 10.78±1.25 | 0.646 |
| Reticulocyte percentage (%) | 9.55±4.83 | 9.89±3.73 | 0.835 |
| APTT (s) | 27.02±4.09 | 27.33±3.38 | 0.207 |
| TT (s) | 17.06±1.25 | 17.04±1.15 | 0.986 |
| PT (s) | 11.45±0.83 | 11.29±0.70 | 0.052 |
| Antithrombin III (%) | 88.26±14.26 | 88.24±15.09 | 0.991 |
| FDP (μg/mL) | 2.27±2.30 | 2.03±1.06 | 0.608 |
| Fibrinogen (g/L) | 2.72±0.73 | 2.73±0.65 | 0.720 |
| Total protein (g/L) | 68.99±6.09 | 68.54±5.38 | 0.582 |
| Albumin (g/L) | 39.75±4.01 | 40.11±3.75 | 0.385 |
| Globulin (g/L) | 29.25±3.65 | 28.42±3.01 | 0.060 |
| ALT (U/L) | 38.15±55.87 | 33.31±19.48 | 0.721 |
| AST (U/L) | 27.95±28.79 | 24.81±12.51 | 0.349 |
| ALP (U/L) | 76.61±26.20 | 68.27±18.65 | 0.050 |
| LDH (U/L) | 419.32±119.95 | 417.03±131.45 | 0.864 |
| Total cholesterol (mmol/L) | 4.56±1.02 | 4.79±1.07 | 0.155 |
| TG (mmol/L) | 1.90±1.00 | 2.26±2.75 | 0.878 |
| Total bilirubin (μmol/L) | 12.77±7.07 | 12.94±5.10 | 0.213 |
| Unconjugated bilirubin (μmol/L) | 8.78±6.86 | 8.73±4.64 | 0.240 |
| Lipase (U/L) | 85.75±47.31 | 83.86±40.71 | 0.980 |
| Amylase (U/L) | 60.97±19.08 | 65.43±27.72 | 0.592 |
| Calcium (mmol/L) | 2.39±0.13 | 2.37±0.11 | 0.968 |
| Potassium (mmol/L) | 4.09±0.36 | 4.09±0.36 | 0.973 |
| Phosphorus (mmol/L) | 1.44±0.27 | 1.32±0.22 | 0.085 |
| Chlorine (mmol/L) | 102.14±3.30 | 102.23±2.83 | 0.798 |
| Magnesium (mmol/L) | 0.85±0.08 | 0.84±0.06 | 0.548 |
| Sodium (mmol/L) | 140.87±3.05 | 140.81±3.10 | 0.924 |
| proBNP (pmol/L) | 9.76±18.39 | 14.39±20.81 | 0.204 |
| Troponin I (ng/ml) | 0.03±0.01 | 0.03±0.01 | 0.594 |
| Troponin T (ng/ml) | 0.01±0.00 | 0.01±0.01 | 0.235 |
| Myoglobin (ng/ml) | 39.23±21.92 | 36.41±17.18 | 0.844 |
| CK-MB isoenzyme (U/L) | 5.91±5.23 | 6.00±6.02 | 0.960 |
| PCT (μg/L) | 0.05±0.03 | 0.05±0.03 | 0.802 |
| CRP (mg/L) | 2.62±7.77 | 2.42±7.24 | 0.284 |
| D-dimer (mg/L) | 0.37±0.66 | 0.27±0.29 | 0.321 |
| IL-6 (ng/L) | 5.45±7.55 | 6.60±14.87 | 0.697 |
| INR | 1.00±0.07 | 0.98±0.06 | 0.051 |
| Creatinine (μmol/L) | 61.13±16.22 | 61.46±20.38 | 0.769 |
| Urea (mmol/L) | 5.11±1.31 | 5.29±1.65 | 0.563 |
| Uric acid (μmol/L) | 306.26±81.29 | 301.75±85.63 | 0.806 |
| Glucose (mmol/L) | 5.33±1.98 | 5.60±2.17 | 0.364 |
| Total carbon dioxide (mmol/L) | 26.13±2.79 | 26.48±2.72 | 0.363 |

ACTH secreting, adrenocorticotropic hormone secreting; GH secreting, growth hormone secreting; PRL secreting, prolactin secreting; LH, luteinizing hormone; FSH, follicle-stimulating hormone; DHEAS, dehydroepiandrosterone sulfate; TSH, thyroid-stimulating hormone; T3, triiodothyronine; T4, tetraiodothyronine; FT3, free triiodothyronine; FT4, free tetraiodothyronine; ACTH, adrenocorticotropic hormone; IGF-1, insulin-like growth factor-1; IGFBP3, insulin-like growth factor binding protein 3; GH, growth hormone; RBC, red blood cell; HCT, haematocrit; RDW, red blood cell distribution width; MCV, mean corpuscular volume; MCH, mean corpuscular hemoglobin; MCHC, mean corpuscular hemoglobin concentration; WBC, white blood cell; MPV, mean platelet volume; APTT, activated partial thromboplastin time; TT, thrombin time; PT, prothrombin time; FDP, fibrin/fibrinogen degradation products; ALT, alanine aminotransferase; AST, aspartate transaminase; ALP, alkaline phosphatase; LDH, lactate dehydrogenase; TG, triglyceride; proBNP, pro-brain natriuretic peptide; PCT, procalcitonin; CRP, C-reactive protein; IL-6, interleukin-6; INR, international normalized ratio. *Statistical significance.
