# Supplementary material for: A Nomogram for Preoperatively Predicting the Ki-67 Index of a Pituitary Tumor: A Retrospective Cohort Study
Source: Front Oncol. 2021 May 31;11:687333. doi: 10.3389/fonc.2021.687333 (PMC8200848; doi:10.3389/fonc.2021.687333)
Supplement: Supplementary Table 3 — Other characteristics of patients with PA in the low and high Ki-67 cohorts. [file Table_3.docx]

Supplementary Table 3. Other characteristics of patients with PA in the low and high Ki-67 cohorts

| Characteristics | Low Ki-67 | High Ki-67 | *p* |
| --- | --- | --- | --- |
| Headache |  |  | 0.934 |
| No | 193 (75.7%) | 134 (74.9%) |  |
| Yes | 62 (24.3%) | 45 (25.1%) |  |
| Visual impairment |  |  | 1.000 |
| No | 121 (47.6%) | 86 (48%) |  |
| Yes | 133 (52.4%) | 93 (52%) |  |
| Visual field defect |  |  | 0.316 |
| No | 191 (75.2%) | 126 (70.4%) |  |
| Yes | 63 (24.8%) | 53 (29.6%) |  |
| Moon face |  |  | 0.535 |
| No | 248 (97.3%) | 176 (98.3%) |  |
| Yes | 7 (2.7%) | 3 (1.7%) |  |
| Acromegalia |  |  | 0.223 |
| No | 210 (82.4%) | 156 (87.2%) |  |
| Yes | 45 (17.6%) | 23 (12.8%) |  |
| Optic nerve compression |  |  | 0.127 |
| No | 79 (36.9%) | 38 (28.4%) |  |
| Yes | 135 (63.1%) | 96 (71.6%) |  |
| Pituitary apoplexy |  |  | 0.890 |
| No | 151 (70.6%) | 97 (71.9%) |  |
| Yes | 63 (29.4%) | 38 (28.1%) |  |
| History of medication |  |  | 0.942 |
| No | 242 (94.9%) | 171 (95.5%) |  |
| Yes | 13 (5.1%) | 8 (4.5%) |  |
| History of radiotherapy |  |  | 0.705 |
| No | 250 (98%) | 177 (98.9%) |  |
| Yes | 5 (2%) | 2 (1.1%) |  |
| Testosterone (nmol/L) | 4.33±5.16 | 4.53±5.16 | 0.569 |
| Estradiol (pmol/L) | 169.08±217.47 | 166.31±243.57 | 0.706 |
| Progesterone (nmol/L) | 2.19±4.02 | 3.35±8.86 | 0.941 |
| DHEAS (umol/L) | 3.65±2.68 | 3.99±3.08 | 0.544 |
| TSH (mIU/L) | 2.04±1.71 | 2.39±3.54 | 0.451 |
| T4 (nmol/L) | 99.54±25.53 | 96.42±27.68 | 0.052 |
| FT4 (pmol/L) | 9.64±2.52 | 9.54±3.37 | 0.115 |
| ACTH (pg/ml) | 28.44±31.30 | 29.01±25.58 | 0.611 |
| Cortisol (μmol/L) | 0.29±0.15 | 0.28±0.14 | 0.903 |
| IGF-1 (ng/ml) | 348.33±326.50 | 175.45±161.36 | 0.072 |
| IGFBP3 (mg/L) | 5.11±2.36 | 5.24±2.18 | 0.412 |
| GH (μg/L) | 3.98±9.20 | 3.25±8.03 | 0.358 |
| HCT (%) | 0.39±0.04 | 0.39±0.04 | 0.890 |
| RDW (%) | 12.99±1.24 | 13.06±1.25 | 0.894 |
| Hemoglobin (g/L) | 130.81±15.38 | 130.31±15.53 | 0.694 |
| MCHC (g/L) | 335.67±12.23 | 333.82±13.37 | 0.097 |
| WBC count (10^9^/L) | 5.51±1.64 | 5.50±1.44 | 0.790 |
| Neutrophil percentage (%) | 54.53±9.79 | 52.91±9.45 | 0.088 |
| Monocyte percentage (%) | 7.48±1.83 | 7.58±1.95 | 0.714 |
| Basophil percentage (%) | 0.42±0.28 | 0.41±0.22 | 0.909 |
| Eosinophil percentage (%) | 2.72±2.24 | 2.49±1.65 | 0.932 |
| MPV (fL) | 10.90±1.33 | 10.76±1.31 | 0.225 |
| Reticulocyte percentage (%) | 8.78±4.01 | 10.82±5.01 | 0.299 |
| APTT (s) | 27.18±3.89 | 27.02±3.88 | 0.957 |
| TT (s) | 17.08±1.24 | 17.04±1.19 | 0.784 |
| PT (s) | 11.41±0.83 | 11.38±0.74 | 0.979 |
| Antithrombin III (%) | 88.59±15.18 | 87.86±13.80 | 0.693 |
| Fibrinogen (g/L) | 2.74±0.71 | 2.70±0.69 | 0.630 |
| Total protein (g/L) | 68.85±5.81 | 68.82±6.04 | 0.870 |
| Albumin (g/L) | 39.65±3.94 | 40.16±3.95 | 0.208 |
| Globulin (g/L) | 29.21±3.48 | 28.66±3.47 | 0.133 |
| ALT (U/L) | 35.24±23.49 | 38.56±68.45 | 0.628 |
| AST (U/L) | 26.98±17.10 | 26.94±32.98 | 0.246 |
| ALP (U/L) | 74.17±22.99 | 73.09±26.42 | 0.731 |
| LDH (U/L) | 435.12±119.14 | 391.43±126.58 | 0.051 |
| Total cholesterol (mmol/L) | 4.64±1.06 | 4.63±1.00 | 0.755 |
| TG (mmol/L) | 2.03±2.05 | 2.00±0.98 | 0.159 |
| Total bilirubin (μmol/L) | 13.08±6.50 | 12.46±6.50 | 0.292 |
| Unconjugated bilirubin (μmol/L) | 8.88±6.28 | 8.56±6.21 | 0.514 |
| Lipase (U/L) | 83.14±43.44 | 89.44±49.13 | 0.791 |
| Amylase (U/L) | 61.37±22.38 | 64.57±21.69 | 0.655 |
| Calcium (mmol/L) | 2.39±0.11 | 2.38±0.15 | 0.764 |
| Phosphorus (mmol/L) | 1.36±0.25 | 1.47±0.27 | 0.170 |
| Chlorine (mmol/L) | 102.15±3.20 | 102.16±3.11 | 0.787 |
| Magnesium (mmol/L) | 0.85±0.07 | 0.84±0.07 | 0.756 |
| Sodium (mmol/L) | 141.04±3.29 | 140.57±2.73 | 0.074 |
| proBNP (pmol/L) | 11.55±18.49 | 10.31±21.32 | 0.387 |
| Troponin I (ng/ml) | 0.03±0.01 | 0.03±0.01 | 0.226 |
| Troponin T (ng/ml) | 0.01±0.00 | 0.01±0.01 | 0.208 |
| Myoglobin (ng/ml) | 37.67±20.71 | 40.53±20.57 | 0.762 |
| CK-MB isoenzyme (U/L) | 5.94±5.80 | 5.96±5.00 | 0.829 |
| D-dimer (mg/L) | 0.36±0.64 | 0.30±0.44 | 0.820 |
| IL-6 (ng/L) | 6.82±12.84 | 3.81±2.68 | 0.052 |
| INR | 0.99±0.07 | 0.99±0.06 | 0.977 |
| Uric acid (μmol/L) | 302.69±82.15 | 309.37±83.80 | 0.753 |
| Glucose (mmol/L) | 5.53±2.12 | 5.27±1.95 | 0.366 |
| Total carbon dioxide (mmol/L) | 26.42±2.74 | 26.01±2.83 | 0.289 |

DHEAS, dehydroepiandrosterone sulfate; TSH, thyroid-stimulating hormone; T4, tetraiodothyronine; FT4, free tetraiodothyronine; ACTH, adrenocorticotropic hormone; IGF-1, insulin-like growth factor-1; IGFBP3, insulin-like growth factor binding protein 3; GH, growth hormone; HCT, haematocrit; RDW, red blood cell distribution width; MCHC, mean corpuscular hemoglobin concentration; WBC, white blood cell; MPV, mean platelet volume; APTT, activated partial thromboplastin time; TT, thrombin time; PT, prothrombin time; ALT, alanine aminotransferase; AST, aspartate transaminase; ALP, alkaline phosphatase; LDH, lactate dehydrogenase; TG, triglyceride; proBNP, pro-brain natriuretic peptide; IL-6, interleukin-6; INR, international normalized ratio.
