# Supplementary material for: A Nomogram for Preoperatively Predicting the Ki-67 Index of a Pituitary Tumor: A Retrospective Cohort Study
Source: Front Oncol. 2021 May 31;11:687333. doi: 10.3389/fonc.2021.687333 (PMC8200848; doi:10.3389/fonc.2021.687333)
Supplement: Supplementary Table 4 — Univariate logistic regression analysis of features in the training cohort. [file Table_4.docx]

Supplementary Table 4. Univariate logistic regression analysis of features in the training cohort

| Characteristics | Coefficient | OR | *p* |
| --- | --- | --- | --- |
| Age (year) | –0.0401 | 0.9607 | <0.0001* |
| Gender |  |  |  |
| Female | Reference |  |  |
| Male | 0.1217 | 1.1294 | 0.6037 |
| Clinical subtype |  |  |  |
| Nonfunctioning | Reference |  |  |
| PRL secreting | 1.4042 | 4.0723 | 0.0012* |
| GH secreting | –0.2029 | 0.8164 | 0.4676 |
| ACTH secreting | 0.2549 | 1.2903 | 0.8002 |
| Primary-recurrence subtype |  |  |  |
| Primary | Reference |  |  |
| Recurrence | 0.7671 | 2.1535 | 0.0189* |
| Maximum dimension (mm) | 0.0358 | 1.0364 | 0.0020* |
| Knosp grade |  |  |  |
| Noninvasive | Reference |  |  |
| Invasive | 0.3960 | 1.4859 | 0.1345 |
| Hardy grade for suprasellar extension | |  |  |
| 0 | Reference |  |  |
| A | 0.3441 | 1.4107 | 0.2895 |
| B | 0.0847 | 1.0884 | 0.7707 |
| C | –0.3348 | 0.7155 | 0.3279 |
| D | 1.6692 | 5.3079 | 0.0401* |
| E | 2.6431 | 14.0567 | 0.0129* |
| Hardy grade for sellar invasion |  |  |  |
| Noninvasive | Reference |  |  |
| Invasive | 0.2055 | 1.2281 | 0.4803 |
| Multiple lesions |  |  |  |
| No | Reference |  |  |
| Yes | 2.0113 | 7.4730 | 0.0684 |
| Optic nerve compression |  |  |  |
| No | Reference |  |  |
| Yes | 0.5532 | 1.7388 | 0.0541 |
| Pituitary apoplexy |  |  |  |
| No | Reference |  |  |
| Yes | 0.0285 | 1.0289 | 0.9241 |
| Headache |  |  |  |
| No | Reference |  |  |
| Yes | 0.0632 | 1.0652 | 0.8184 |
| Visual impairment |  |  |  |
| No | Reference |  |  |
| Yes | 0.0224 | 1.0227 | 0.9242 |
| Visual field defect |  |  |  |
| No | Reference |  |  |
| Yes | 0.2787 | 1.3214 | 0.2839 |
| Moon face |  |  |  |
| No | Reference |  |  |
| Yes | –0.4193 | 0.6575 | 0.7332 |
| Acromegalia |  |  |  |
| No | Reference |  |  |
| Yes | –0.5033 | 0.6045 | 0.1114 |
| History of pituitary surgery |  |  |  |
| No | Reference |  |  |
| Yes | 0.7689 | 2.1574 | 0.014* |
| History of medication |  |  |  |
| No | Reference |  |  |
| Yes | 0.1521 | 1.1643 | 0.7747 |
| History of radiotherapy |  |  |  |
| No | Reference |  |  |
| Yes | –0.8308 | 0.4357 | 0.4741 |
| Prolacin (mIU/L) | 0.0005 | 1.0005 | 0.0027* |
| Testosterone (nmol/L) | 0.0011 | 1.0011 | 0.9657 |
| Estradiol (pmol/L) | 0.0005 | 1.0005 | 0.3900 |
| Progesterone (nmol/L) | 0.0341 | 1.0347 | 0.1422 |
| LH (IU/L) | –0.0788 | 0.9242 | 0.0013* |
| FSH (IU/L) | –0.0358 | 0.9648 | 0.0003* |
| DHEAS (umol/L) | 0.0493 | 1.0505 | 0.2820 |
| TSH (mIU/L) | 0.0482 | 1.0494 | 0.3194 |
| T3 (nmol/L) | 0.8091 | 2.2459 | 0.0348* |
| T4 (nmol/L) | –0.0054 | 0.9946 | 0.2445 |
| FT3 (pmol/L) | 0.4660 | 1.5936 | 0.0045* |
| FT4 (pmol/L) | –0.0315 | 0.9690 | 0.4689 |
| ACTH (pg/ml) | 0.0038 | 1.0038 | 0.6217 |
| Cortisol (μmol/L) | –0.6670 | 0.5132 | 0.4270 |
| IGF-1 (ng/ml) | –0.0034 | 0.9966 | 0.0547 |
| IGFBP3 (mg/L) | 0.0025 | 1.0025 | 0.9692 |
| GH (μg/L) | –0.0198 | 0.9804 | 0.1622 |
| RBC count (10^12^/L) | 0.5418 | 1.7191 | 0.0251* |
| HCT (%) | 2.2310 | 9.3092 | 0.4061 |
| RDW (%) | 0.0581 | 1.0598 | 0.4884 |
| MCV (fL) | –0.0507 | 0.9506 | 0.0175* |
| MCH (pg) | –0.1435 | 0.8663 | 0.0091* |
| Hemoglobin (g/L) | 0.0029 | 1.0029 | 0.6933 |
| MCHC (g/L) | –0.0120 | 0.9881 | 0.1911 |
| WBC count (10^9^/L) | 0.1110 | 1.1174 | 0.1745 |
| Neutrophil percentage (%) | –0.0181 | 0.9821 | 0.1553 |
| Lymphocyte percentage (%) | 0.0268 | 1.0272 | 0.0510 |
| Monocyte percentage (%) | –0.0097 | 0.9903 | 0.8776 |
| Basophil percentage (%) | –0.4740 | 0.6225 | 0.3034 |
| Eosinophil percentage (%) | –0.0894 | 0.9145 | 0.1334 |
| Platelet count (10^9/L) | 0.0043 | 1.0043 | 0.0286* |
| Thrombocytocrit (%) | 4.6330 | 102.8221 | 0.0251* |
| MPV (fL) | –0.0726 | 0.9300 | 0.4167 |
| Reticulocyte percentage (%) | 0.1041 | 1.1097 | 0.2709 |
| APTT (s) | –0.0200 | 0.9802 | 0.5042 |
| TT (s) | 0.0048 | 1.0048 | 0.9611 |
| PT (s) | –0.1933 | 0.8242 | 0.2019 |
| Antithrombin III (%) | –0.0061 | 0.9939 | 0.5761 |
| FDP (μg/mL) | –0.1035 | 0.9017 | 0.2546 |
| Fibrinogen (g/L) | –0.0677 | 0.9345 | 0.6901 |
| Total protein (g/L) | 0.0122 | 1.0123 | 0.5447 |
| Albumin (g/L) | 0.0579 | 1.0596 | 0.0642 |
| Globulin (g/L) | –0.0365 | 0.9642 | 0.2865 |
| ALT (U/L) | 0.0018 | 1.0018 | 0.4634 |
| AST (U/L) | 0.0020 | 1.0020 | 0.6629 |
| ALP (U/L) | 0.0032 | 1.0032 | 0.6496 |
| LDH (U/L) | –0.0006 | 0.9994 | 0.7626 |
| Total cholesterol (mmol/L) | 0.1793 | 1.1964 | 0.3586 |
| TG (mmol/L) | 0.3857 | 1.4706 | 0.0610 |
| Total bilirubin (μmol/L) | –0.0098 | 0.9902 | 0.6223 |
| Unconjugated bilirubin (μmol/L) | –0.0094 | 0.9906 | 0.6611 |
| Lipase (U/L) | –0.0020 | 0.9980 | 0.8135 |
| Amylase (U/L) | –0.0033 | 0.9967 | 0.8743 |
| Calcium (mmol/L) | –0.1371 | 0.8719 | 0.9423 |
| Potassium (mmol/L) | 1.0788 | 2.9411 | 0.0019* |
| Phosphorus (mmol/L) | 1.3649 | 3.9153 | 0.3238 |
| Chlorine (mmol/L) | –0.0061 | 0.9939 | 0.8664 |
| Magnesium (mmol/L) | –3.2335 | 0.0394 | 0.5088 |
| Sodium (mmol/L) | –0.0443 | 0.9567 | 0.2612 |
| proBNP (pmol/L) | 0.0021 | 1.0021 | 0.9162 |
| Troponin I (ng/ml) | 51.7083 | 2.86*10^12^ | 0.2048 |
| Troponin T (ng/ml) | 123.3808 | 3.83*10^53^ | 0.2066 |
| Myoglobin (ng/ml) | 0.0121 | 1.0122 | 0.4570 |
| CK-MB isoenzyme (U/L) | 0.0057 | 1.0057 | 0.9215 |
| PCT (μg/L) | –27.7079 | 0.0000 | 0.0068* |
| CRP (mg/L) | –0.0221 | 0.9781 | 0.3218 |
| D-dimer (mg/L) | –0.2166 | 0.8053 | 0.4287 |
| IL-6 (ng/L) | –0.1223 | 0.8849 | 0.0511 |
| INR | –2.3113 | 0.0991 | 0.1891 |
| Creatinine (μmol/L) | –0.0029 | 0.9971 | 0.7006 |
| Urea (mmol/L) | –0.1703 | 0.8434 | 0.0699 |
| Uric acid (μmol/L) | 0.0017 | 1.0017 | 0.2952 |
| Glucose (mmol/L) | –0.1058 | 0.8996 | 0.2577 |
| Total carbon dioxide (mmol/L) | –0.0836 | 0.9198 | 0.1602 |

OR, odds ratio; ACTH secreting, adrenocorticotropic hormone secreting; GH secreting, growth hormone secreting; PRL secreting, prolactin secreting; LH, luteinizing hormone; FSH, follicle-stimulating hormone; DHEAS, dehydroepiandrosterone sulfate; TSH, thyroid-stimulating hormone; T3, triiodothyronine; T4, tetraiodothyronine; FT3, free triiodothyronine; FT4, free tetraiodothyronine; ACTH, adrenocorticotropic hormone; IGF-1, insulin-like growth factor-1; IGFBP3, insulin-like growth factor binding protein 3; GH, growth hormone; RBC, red blood cell; HCT, haematocrit; RDW, red blood cell distribution width; MCV, mean corpuscular volume; MCH, mean corpuscular hemoglobin; MCHC, mean corpuscular hemoglobin concentration; WBC, white blood cell; MPV, mean platelet volume; APTT, activated partial thromboplastin time; TT, thrombin time; PT, prothrombin time; FDP, fibrin/fibrinogen degradation products; ALT, alanine aminotransferase; AST, aspartate transaminase; ALP, alkaline phosphatase; LDH, lactate dehydrogenase; TG, triglyceride; proBNP, pro-brain natriuretic peptide; PCT, procalcitonin; CRP, C-reactive protein; IL-6, interleukin-6; INR, international normalized ratio. *Statistical significance.
