# Supplementary material for: A Nomogram for Preoperatively Predicting the Ki-67 Index of a Pituitary Tumor: A Retrospective Cohort Study
Source: Front Oncol. 2021 May 31;11:687333. doi: 10.3389/fonc.2021.687333 (PMC8200848; doi:10.3389/fonc.2021.687333)
Supplement: Supplementary Table 5 — LASSO analysis in the training cohort. [file Table_5.docx]

Supplementary Table 5. LASSO analysis in the training cohort

| Characteristics | Coefficient |
| --- | --- |
| Intercept | –1.6704655 |
| Age (year) | –0.0128274 |
| Gender |  |
| Female | Reference |
| Male | 0 |
| Clinical subtype |  |
| Nonfunctioning | Reference |
| PRL secreting | 0.2734138 |
| GH secreting | 0 |
| ACTH secreting | 0 |
| Primary-recurrence subtype |  |
| Primary | Reference |
| Recurrence | 0.04803813 |
| Maximum dimension (mm) | 0.01425001 |
| Knosp grade |  |
| Noninvasive | Reference |
| Invasive | 0 |
| Hardy grade for suprasellar extension | |
| 0 | Reference |
| A | 0 |
| B | 0 |
| C | 0 |
| D | 0 |
| E | 0.63201764 |
| Hardy grade for sellar invasion |  |
| Noninvasive | Reference |
| Invasive | 0 |
| Multiple lesions |  |
| No | Reference |
| Yes | 0 |
| Optic nerve compression |  |
| No | Reference |
| Yes | 0 |
| Pituitary apoplexy |  |
| No | Reference |
| Yes | 0 |
| Headache |  |
| No | Reference |
| Yes | 0 |
| Visual impairment |  |
| No | Reference |
| Yes | 0 |
| Visual field defect |  |
| No | Reference |
| Yes | 0 |
| Moon face |  |
| No | Reference |
| Yes | 0 |
| Acromegalia |  |
| No | Reference |
| Yes | 0 |
| History of pituitary surgery |  |
| No | Reference |
| Yes | 0 |
| History of medication |  |
| No | Reference |
| Yes | 0 |
| History of radiotherapy |  |
| No | Reference |
| Yes | 0 |
| Prolacin (mIU/L) | 0 |
| Testosterone (nmol/L) | 0 |
| Estradiol (pmol/L) | 0 |
| Progesterone (nmol/L) | 0 |
| LH (IU/L) | –0.0034925 |
| FSH (IU/L) | –0.013025 |
| DHEAS (umol/L) | 0 |
| TSH (mIU/L) | 0 |
| T3 (nmol/L) | 0 |
| T4 (nmol/L) | 0 |
| FT3 (pmol/L) | 0.14609309 |
| FT4 (pmol/L) | 0 |
| ACTH (pg/ml) | 0 |
| Cortisol (μmol/L) | 0 |
| IGF-1 (ng/ml) | 0 |
| GH (μg/L) | 0 |
| RBC count (10^12^/L) | 0 |
| HCT (%) | 0 |
| RDW (%) | 0 |
| MCV (fL) | 0 |
| MCH (pg) | –0.0162112 |
| Hemoglobin (g/L) | 0 |
| MCHC (g/L) | 0 |
| WBC count (10^9^/L) | 0 |
| Neutrophil percentage (%) | 0 |
| Lymphocyte percentage (%) | 0 |
| Monocyte percentage (%) | 0 |
| Basophil percentage (%) | 0 |
| Eosinophil percentage (%) | 0 |
| Platelet count (10^9^/L) | 0 |
| Thrombocytocrit (%) | 0 |
| MPV (fL) | 0 |
| APTT (s) | 0 |
| TT (s) | 0 |
| PT (s) | 0 |
| Antithrombin III (%) | 0 |
| FDP (μg/mL) | 0 |
| Fibrinogen (g/L) | 0 |
| Total protein (g/L) | 0 |
| Albumin (g/L) | 0 |
| Globulin (g/L) | 0 |
| ALT (U/L) | 0 |
| AST (U/L) | 0 |
| ALP (U/L) | 0 |
| Total bilirubin (μmol/L) | 0 |
| Unconjugated bilirubin (μmol/L) | 0 |
| Potassium (mmol/L) | 0.39848911 |
| Chlorine (mmol/L) | 0 |
| Sodium (mmol/L) | 0 |
| CRP (mg/L) | 0 |
| D-dimer (mg/L) | 0 |
| INR | 0 |
| Creatinine (μmol/L) | 0 |
| Urea (mmol/L) | 0 |
| Uric acid (μmol/L) | 0 |
| Glucose (mmol/L) | 0 |
| Total carbon dioxide (mmol/L) | 0 |

ACTH secreting, adrenocorticotropic hormone secreting; GH secreting, growth hormone secreting; PRL secreting, prolactin secreting; LH, luteinizing hormone; FSH, follicle-stimulating hormone; DHEAS, dehydroepiandrosterone sulfate; TSH, thyroid-stimulating hormone; T3, triiodothyronine; T4, tetraiodothyronine; FT3, free triiodothyronine; FT4, free tetraiodothyronine; ACTH, adrenocorticotropic hormone; IGF-1, insulin-like growth factor-1; GH, growth hormone; RBC, red blood cell; HCT, haematocrit; RDW, red blood cell distribution width; MCV, mean corpuscular volume; MCH, mean corpuscular hemoglobin; MCHC, mean corpuscular hemoglobin concentration; WBC, white blood cell; MPV, mean platelet volume; APTT, activated partial thromboplastin time; TT, thrombin time; PT, prothrombin time; FDP, fibrin/fibrinogen degradation products; ALT, alanine aminotransferase; AST, aspartate transaminase; ALP, alkaline phosphatase; CRP, C-reactive protein; INR, international normalized ratio.
