# Supplementary figures and images for: A Nomogram for Preoperatively Predicting the Ki-67 Index of a Pituitary Tumor: A Retrospective Cohort Study
Source: Front Oncol. 2021 May 31;11:687333. doi: 10.3389/fonc.2021.687333 (PMC8200848; doi:10.3389/fonc.2021.687333)

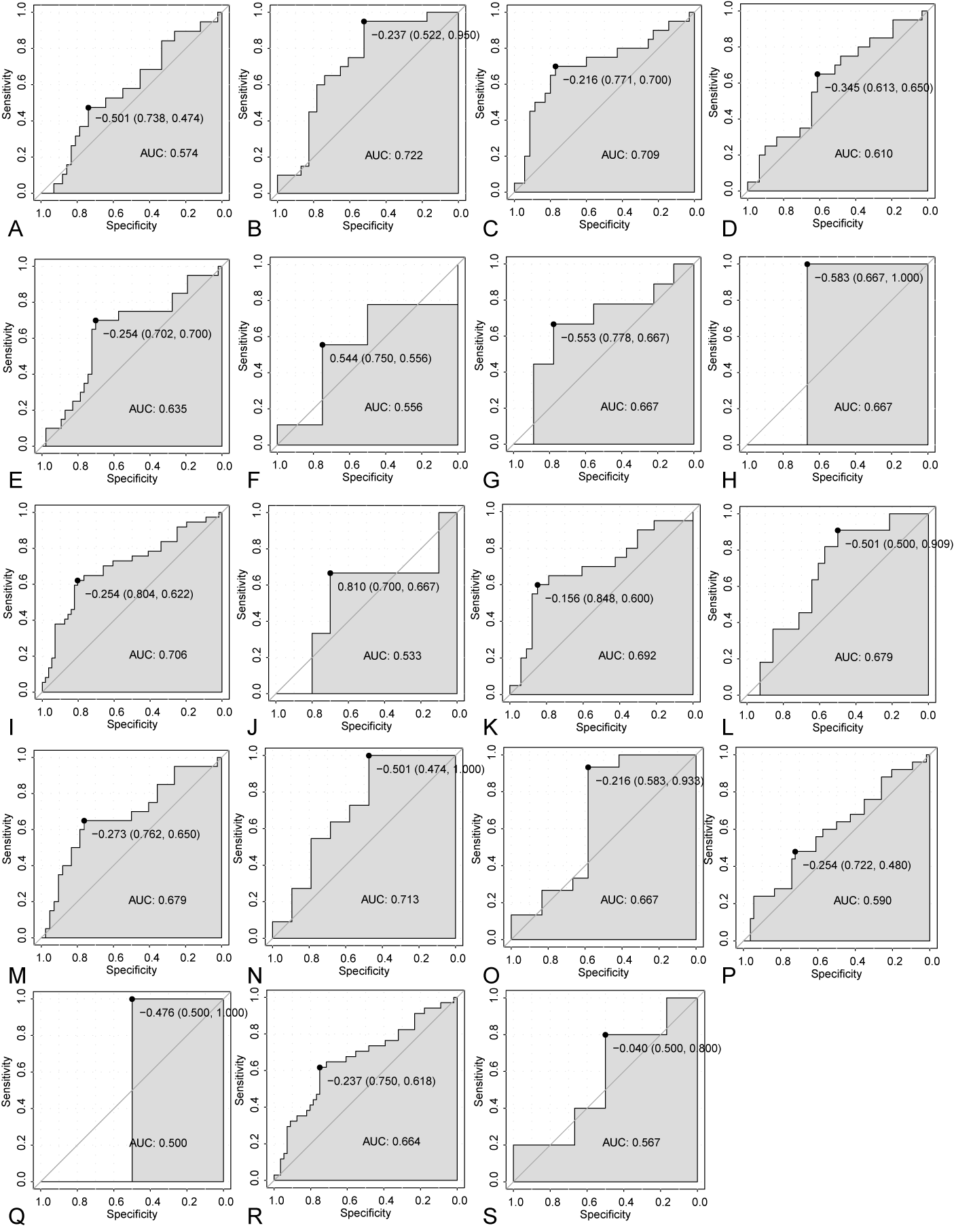

Supplement: Supplementary Figure 1 — Predictive performance for the nomogram in the subgroup analysis. (A, B) ROC analysis in the subgroup according to age (A for >51 years and B for ≤51 years); (C, D) ROC analysis in the subgroup according to gender (C for female and D for male); (E–H) ROC analysis in the subgroup according to clinical subtypes (E for nonfunctioning; F for PRL secreting; G for GH secreting; and H for ACTH secreting pituitary tumors); (I, J) ROC analysis in the subgroup according to primary-recurrence subtypes (I for primary and J for recurrence subtypes); (K, L) ROC analysis in the subgroup according to Knosp grade (K for noninvasive and L for invasive); (M, N) ROC analysis in the subgroup according to Hardy grade (M for noninvasive and N for invasive); (O, P) ROC analysis in the subgroup according to prolactin (O for >815 mIU/L and P for ≤815 mIU/L); (Q–S) ROC analysis in the subgroup according to the maximum dimension (Q for microadenoma, R for macroadenoma, and S for giant adenoma). [file Image_1.tif]

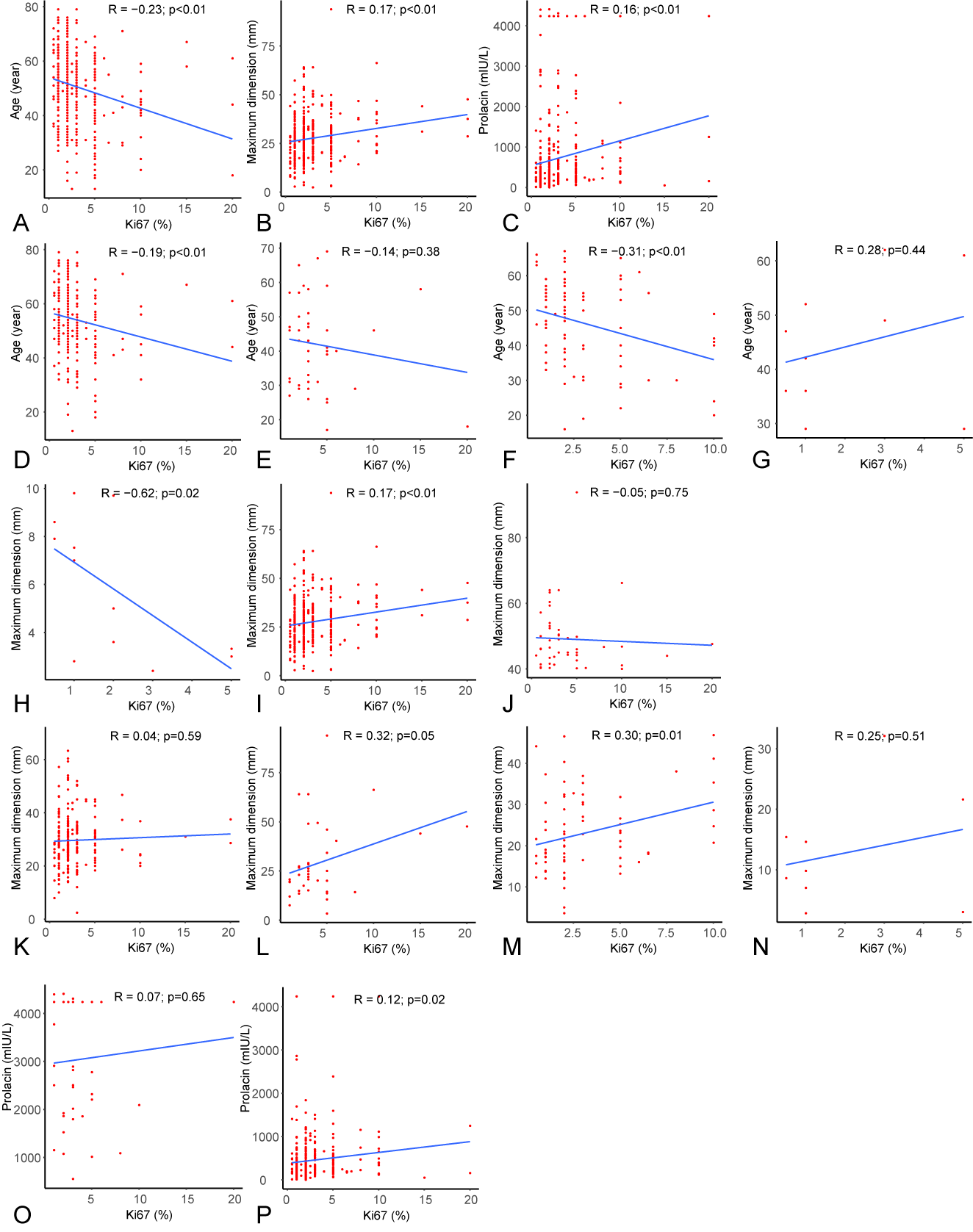

Supplement: Supplementary Figure 2 — Correlation analysis. (A–C) Correlation between Ki-67 index and age (A), maximum dimension (B), and prolactin (C), respectively, in all of the validation cohort. (D–G) Correlation between Ki-67 index and age in the subgroups according to nonfunctioning (D), PRL secreting (E), GH secreting (F), and ACTH secreting (G) pituitary tumors. (H–N) correlation between Ki-67 index and maximum dimension in the subgroups according to microadenoma (H), macroadenoma (I), giant adenoma (J), nonfunctioning (K), PRL secreting (L), GH secreting (M), and ACTH secreting (N) pituitary tumors. (O–P) correlation between Ki-67 index and prolactin in the subgroups according to prolactinoma (O) and no prolactinoma (P). [file Image_2.tif]
